# Synthesis, Antiproliferative Activity, ADME Profiling, and Docking Studies of Novel 1, 2, 3-Triazole Derivatives of 2-Amino and 2-Mercaptobenzoxazole

**DOI:** 10.2174/0109298673393777250714045359

**Published:** 2025-07-31

**Authors:** Robert Ostrički, Anja Rakas, Vesna Rastija, Leentje Persoons, Dirk Daelemans, Tatjana Gazivoda Kraljević

**Affiliations:** 1 Department of Organic Chemistry, Faculty of Chemical Engineering and Technology, University of Zagreb, Marulićev trg 20, 10000, Zagreb, Croatia;; 2 Faculty of Agrobiotechnical Sciences Osijek, Josip Juraj Strossmayer University of Osijek, Vladimira Preloga 1, 31000, Osijek, Croatia;; 3 Department of Microbiology, Immunology and Transplantation, Molecular Genetics and Therapeutics in Virology and Oncology Research Group, Rega Institute for Medical Research, KU Leuven, 3000, Leuven, Belgium;; 4 Department for Packaging, Recycling and Environmental Protection, University North, Trg dr. Žarka Dolinara 1, 48000, Koprivnica, Croatia

**Keywords:** benzoxazole, 1,2,3-triazole, click chemistry, CuAAC reaction, antitumor activity, molecular docking

## Abstract

**Introduction:**

Benzoxazole is a privileged scaffold with diverse biological activities, and its hybridization with a 1,2,3-triazole ring can improve affinity and efficacy. This study aimed to synthesize novel 1,2,3-triazole derivatives of 2-aminobenzoxazole and 2-mercaptobenzoxazole, and to evaluate their antiproliferative activity, predicted pharmacokinetic properties, and molecular interactions with kinase targets.

**Methods:**

1,2,3-triazole derivatives of 2-aminobenzoxazole **3−15** and 2-mercaptobenzoxazole **18−32** were synthesized *via* cyclization, propargylation, and copper-catalyzed click reaction. Antiproliferative activity was evaluated against human cancer cell lines: LN-229, Capan-1, HCT-116, NCI-H460, DND-41, HL-60, K-562, and Z-138. The ADME properties of 1,2,3-triazole-benzoxazole hybrids were evaluated using the SwissADME tool. The most active compounds were assessed for Human Gastrointestinal Absorption (HGA) and Blood-Brain Barrier (BBB) permeability using the Egan model. Molecular docking was performed on serine/threonine kinase TAO2 and tyrosine kinase c-Src.

**Results:**

A series of novel 1,2,3-triazole derivatives of 2-amino **3−15** and 2-mercaptobenzoxazole **18−32** were synthesized *via* click chemistry. Coumarin-containing compounds **3** and **29** showed the most pronounced antiproliferative activity across all tested cell lines. Both demonstrated high predicted HGA and low likelihood of crossing the BBB. Compound **3** exhibited the highest binding affinity for TAO2, while compound **29** showed strong interaction with c-Src.

**Discussion:**

The results highlight the favorable influence of coumarin substitution on antiproliferative activity, with computational ADME and docking data supporting the observed *in vitro* efficacy.

**Conclusion:**

This study outlines a viable method for the synthesis of novel 1,2,3-triazole derivatives of 2-aminobenzoxazole and 2-mercaptobenzoxazole. Compounds **3** and **29** demonstrate promising antiproliferative activity and pharmacokinetic potential, supporting their further development as anticancer candidates.

## 1 INTRODUCTION

**Fig. (1):**
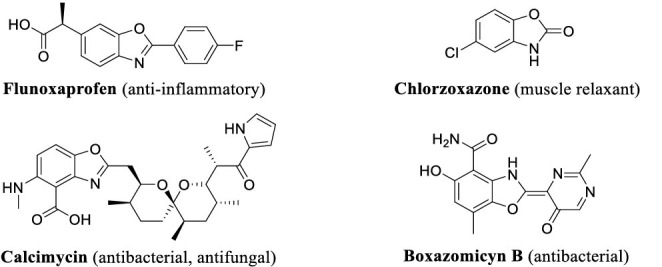
Approved drugs containing the benzoxazole moiety.

Cancer arises from the uncontrolled transformation of normal cells into tumor cells, which can proliferate rapidly and spread throughout the body. Along with cardiovascular diseases, cancer is a major contributor to global mortality, accounting for a significant number of deaths worldwide. According to the World Health Organization, cancer was responsible for nearly 10 million deaths in 2020 alone. The most common types of cancer diagnosed that year were breast cancer (2.26 million cases), lung cancer (2.21 million cases), colorectal cancer (1.93 million cases), prostate cancer (1.41 million cases), skin cancer (1.2 million cases), and stomach cancer (1.09 million cases) [1]. Although chemotherapy remains the primary treatment of cancer, the high incidence and mortality rates, along with the failure of existing chemotherapeutics, underscore the urgent need for the development of new, potent antitumor agents. In recent years, the benzoxazole moiety has garnered significant attention as an intermediate for the synthesis of new derivatives with potential biological applications [2]. Benzoxazoles, being structural isosteres of natural nucleotides, interact with biological macromolecules, leading to a wide range of pharmacological activities, including antitumor [3-11], antibacterial [12-18], antiviral [19, 20], and anti-inflammatory effects [21-25]. Additionally, benzoxazole derivatives have applications in other fields, such as fluorescent [26, 27] and functional materials [28-30]. Several marketed drugs contain a benzoxazole moiety as their main active component, including the Nonsteroidal Anti-Inflammatory Drug (NSAID) flunoxaprofen, the muscle relaxant chlorzoxazone, the antibiotic calcimycin, and the antibacterial agent boxazomicin B (Fig. **1**) [31].

**Fig. (2):**
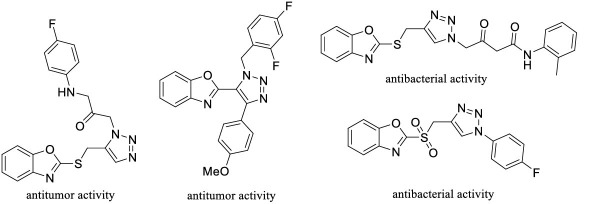
Biologically active 1,2,3-triazole derivatives of benzoxazole.

Molecular hybridization is an effective approach in medicinal chemistry for drug design and development. It involves combining the pharmacophore moieties of different bioactive substances to create a new hybrid compound with improved affinity and efficacy compared to the original components [32]. The 1,2,3-triazole ring is frequently utilized as a pharmacophore in molecular hybridization, resulting in compounds with modified selectivity profiles, reduced unwanted side effects, and distinct or dual modes of action. Consequently, 1,2,3-triazoles have become integral to drug development and discovery, primarily due to their remarkable biological efficacy, ease of synthesis, stability under diverse conditions, and versatility across a wide range of chemical reactions [33, 34]. Given its favorable properties, the 1,2,3-triazole moiety has also been used as a linker between the benzoxazole scaffold and selected pharmacophores. The 1,2,3-triazole moiety serves as a bioisostere of the amide group, which is commonly found in bioactive compounds [35] but can be unstable and prone to hydrolysis. In contrast, the 1,2,3-triazole moiety is stable under various oxidative, reductive, and hydrolytic conditions. Its significant dipole moment allows for the polarization of the H-C-5 bond, enabling hydrogen donation, while the nitrogen atoms at positions 2 and 3 act as hydrogen bond acceptors and coordinate with metal ions. These features positively influence solubility and target binding [36]. The 1,2,3-triazole derivatives primarily exist as 1,4- and 1,5-disubstituted regioisomers. The 1,4-disubstituted isomer is preferred because the 1,5-disubstituted form can be oxidized to an *N*-oxide [37, 38]. Moreover, the 1,4-disubstituted isomer serves as a bioisostere of *trans*-amides, which are more common in nature than *cis*-amides due to reduced steric repulsion between substituents in the *trans*-configuration. This has a favorable impact on binding to biological targets [39]. Various biological activities, such as antitumor [40-43], antimicrobial [44-47], anticonvulsive [48-50], and antianemic [51] effects, have been attributed to 1,2,3-triazole derivatives. Linking the pharmacophoric benzoxazole and 1,2,3-triazole rings, either directly or through a linker, produces compounds exhibiting both antitumor and antibacterial activities (Fig. **2**) [2, 52].

One of the most commonly used methods for preparing the 1,2,3-triazole ring is the Huisgen 1,3-dipolar cycloaddition reaction between terminal alkynes and azides. Initially developed by R. Huisgen, the original reaction required high temperatures and produced a mixture of 1,4- and 1,5-regioisomers [53]. Sharpless and Meldal [54, 55] later enhanced this concept by introducing Copper(I) as a catalyst, which selectively yields the 1,4-regioisomer at room temperature in aqueous conditions, making copper-catalyzed azide-alkyne cycloaddition (CuAAC) synonymous with click chemistry. This groundbreaking advancement, which received the Nobel prize in 2022, ushered in the era of functionalism in chemistry and laid the foundations for click chemistry.

To be effective, drugs must reach their target in sufficient concentrations, remain active for an adequate duration, and exhibit high biological activity with low toxicity. Therefore, the Absorption, Distribution, Metabolism, and Excretion (ADME) properties of compounds with antiproliferative activity can be assessed using *in silico* methods [56].

Tyrosine-protein kinases catalyze the transfer of a phosphate group from adenosine triphosphate (ATP) to a tyrosine residue in a protein. Many steps in neoplastic development and progression involve tyrosine kinases. As a result of tyrosine kinase signaling pathways, the proliferation of cells is normally regulated, or apoptosis is sensitively triggered. Oncogenic cells often alter these signaling pathways to gain a selective advantage. Cytoplasmic/non-receptor tyrosine kinases (C/NRTKs), such as the non-receptor protein tyrosine kinase c-Src, play a crucial role in tumor invasion and progression, making them a promising target for cancer therapy [57]. The activation of c-Src kinases is associated with hematologic malignancies and many solid malignancies, such as breast and colon cancer. Selective tyrosine kinase inhibitors can block the oncogenic activation of these kinases in cancer cells. Thus, c-SRC inhibitors such as imatinib and bosutinib are cytotoxic toward K562 cells, inducing apoptosis during the G2/M phase of the cell cycle, and are used in the treatment of non-Hodgkin lymphoma and leukemia [58]. The Mitogen-Activated Protein Kinase (MAPK) pathway is a widely studied signaling system in eukaryotes, activated by MAPK kinases. The thousand-and-one amino acid 2 (TAO2) kinase is a MAP3K-level kinase that phosphorylates and activates MAP2Ks, MEK3 and MEK6, which in turn activate p38 MAPKs. p38 MAPKs regulate the expression of inflammatory cytokines, including tumor necrosis factor, making MAP3K TAO2 a potential drug target [59].

In this study, a molecular hybridization approach was employed, combining bioactive scaffolds to design and synthesize novel 1,2,3-triazole-linked benzoxazole derivatives. The aim was to explore their *in vitro* antiproliferative activities and ADME properties. To explore the possible mechanisms underlying the antiproliferative activity of the compounds and to identify key interactions with the binding sites of both enzymes, molecular docking studies were performed.

## 2 MATERIALS AND METHODS

All chemicals and solvents for the synthesis of compounds were purchased from commercial suppliers, including Sigma-Aldrich and Tokyo Chemical Industry Co., Ltd., and were used without further purification. For the proliferation assays, the DND-41 cell line was acquired from the Deutsche Sammlung von Mikroorganismen und Zellkulturen (DSMZ Leibniz-Institut, Braunschweig, Germany), while the American Type Culture Collection (ATCC, Manassas, VA, USA) provided the following cancer cell lines: Capan-1, HCT-116, NCI-H460, LN-229, HL-60, K-562, and Z-138. Culture media were purchased from Gibco (Gibco Life Technologies, Merelbeke, Belgium) and supplemented with 10% fetal bovine serum (HyClone, Cytiva, Marlborough, MA, USA).

The CellTiter 96^®^ AQueous Non-Radioactive Cell Proliferation Assay (Promega, Madison, WI, USA) was employed to assess the number of viable cells in proliferation assays. Absorbance was measured using a SpectraMax Plus 384 plate reader (Molecular Devices, San Jose, CA, USA).

Reaction progress was monitored by Ultra-High Performance Liquid Chromatography (UHPLC) using an Agilent Technologies 1290 Infinity II.

^1^H- and ^13^C-NMR spectra were recorded on a Bruker Avance AV 400 spectrometer (5 mm RT direct probehead) using standard Bruker pulse sequences. DMSO-*d_6_* was used as the solvent, and tetramethylsilane (TMS) served as the internal standard. All spectra were acquired at 298K unless otherwise stated. For samples with very low solubility, a few drops of trifluoroacetic acid were added to improve dissolution.

Mass spectra (m/z) were recorded using an Agilent Technologies 1290 Infinity II coupled with an Agilent Technologies 6120 Quadrupole LC/MS mass spectrometer. Melting points were determined using a Büchi Melting Point B-540 apparatus.

Flash chromatography was carried out using an Interchim Puri Flash 430. The initial solvent system consisted of ethyl acetate/hexane (30% EtOAc), and a linear gradient was applied over 10 column volumes, gradually increasing the EtOAc content to 80%, which became the final solvent composition.

## 3 EXPERIMENTAL

### 3.1 Chemical Synthesis

#### 3.1.1 Benzo[d]oxazol-2-amine 1 

2-Aminophenol (5.0 g, 45.8 mmol) and di(imidazole-1-yl)methaneimine (13.3 g, 82.5 mmol) were dissolved in anhydrous tetrahydrofuran (THF) (100 mL). The reaction mixture was stirred under reflux for 3 hours. After completion, the mixture was evaporated to dryness, and the residue was purified by flash chromatography using EtOAc/hexane (30-80%) as the eluent [60, 61]. Compound **1** was obtained as a white powder (5.2 g, 84.7%). ^1^H-NMR (400 MHz, DMSO-*d_6_*) δ 7.34 (2H, s, NH_2_), 7.29 (1H, m, H-4), 7.18 (1H, m, H-7), 7.07 (1H, td, *J* = 7.71, 1.16, H-5), 6.94 (1H, td, *J* = 7.57, 1.33, H-6) ppm. ^13^C-NMR (100 MHz, DMSO-*d_6_*) δ 162.72 (C-2), 147.94 (C-7a), 143.63 (C-3a), 123.42 (C-5), 119.93 (C-6), 115.28 (C-4), 108.39 (107) ppm [MH^+^ = 135.^4^].

#### 3.1.2 N-(Prop-2-yn-1-yl)benzo[d]oxazol-2-amine 2

Compound **1** (4.5 g, 33.6 mmol) was dissolved in DMF (135 mL). To the obtained pink solution, propargyl bromide (167.9 mmol, 18.2 mL) was added dropwise. The reaction mixture was heated at 60°C for 25 hours. Upon completion (monitored by UPLC), the volume was reduced under reduced pressure, leading to the formation of a precipitate. Dichloromethane (DCM) (20 mL) was added to the resulting suspension, and the mixture was stirred for 1hour at room temperature (RT). The precipitate was filtered off, washed with DCM (2 × 10 mL), and dried at 50°C under 10 bar for 8 hours. Compound **2** was obtained as a yellowish solid (4.16 g, 72%, mp 193−195°C). ^1^H-NMR (400 MHz, DMSO-*d_6_*) δ 7.75 (1H, d, *J =* 8.0 Hz, H-4), 7.68 (1H, d, *J* = 7.86 Hz, H-7), 7.50 (1H, td, *J* = 7.89, 0.70 Hz, H-5), 7.42 (1H, td, *J* = 8.02, 0.93 Hz, H-6), 5.17 (2H, d, *J* = 2.42 Hz, CH_2_), 3.71 (1H, t, *J* = 2.40 Hz, CH) ppm. ^13^C-NMR (100 MHz, DMSO-*d_6_*) δ 157.98 (C-2), 143.92 (C-7a), 129.35 (C-3a), 126.09 (C-5), 125.04 (C-6), 111.55 (C-4), 111.26 (C-7), 77.92 (C-3'), 75.28 (C-4'), 33.41 (C-2') ppm [MH^+^ = 173.^5^].

#### 3.1.3 General Procedure for the Synthesis of 1,2,3-triazole derivatives of 2-aminobenzoxazole 3−15

A mixture of compound **2** (1 eq.), the appropriate azide (1.1 eq.), CuSO_4_ x 5 H_2_O (0.1 eq., added as a 0.67 M aqueous solution), sodium ascorbate (0.5 eq.), and a 1:1 mixture of *t*-BuOH/water was stirred at 70°C. After completion of the reaction (monitored by UHPLC), water was added dropwise, resulting in the formation of a precipitate. The obtained suspension was stirred for 1 hour at RT, then filtered. The precipitate was washed with *t*-BuOH/water (1:1) and DCM, and dried at 50°C under 10 mbar for 8 hours. The products were isolated by precipitation or by a combination of extraction and evaporation.

##### 3.1.3.1 4-(4-((benzo[d]oxazol-2-ylamino)methyl)- 1H-1,2,3-triazol-1-yl)-2H-chromen-2-one 3

Compound **3** was prepared according to the general procedure using compound **2** (100 mg, 0.58 mmol), 4-azidocoumarin (120 mg, 0.64 mmol), CuSO_4_ x 5 H_2_O (86.5 µL, 0.058 mmol), sodium ascorbate (58 mg, 0.29 mmol), and *t*-BuOH/water (10 mL). The reaction mixture was stirred overnight at 70°C. After completion, water (14 mL) was added dropwise, and the resulting suspension was stirred for 1 hour at RT. The precipitate was filtered off, washed with *t*-BuOH/water (4 mL) and DCM (5 mL), and dried. Compound **3** was obtained as a yellowish solid (25 mg, 11.9%, mp 240−242°C). ^1^H-NMR (400 MHz, DMSO-*d_6_*) δ 8.88 (1H, s, H-triaz), 7.76 (2H, m, H-4,7), 7.53 (1H, d, *J* = 8.6 Hz, H-5), 7.41 (1H, m, H-6), 7.16 (3H, m, H-7',8',9'), 7.02 (1H, m, H-10'), 6.86 (1H, s, H-13'), 5,28 (2H, s, CH_2_) ppm [MH^+^ = 360.0^2^].

##### 3.1.3.2 4-(4-((benzo[d]oxazol-2-ylamino)methyl)- 1H-1,2,3-triazol-1-yl)-2-hydroxybenzoic acid 4

Compound **4** was prepared according to the general procedure using compound **2** (110 mg, 0.64 mmol), 4-azidosalicylic acid (104 mg, 0.58 mmol), CuSO_4_ x 5 H_2_O (86.5 µL, 0.058 mmol), sodium ascorbate (58 mg, 0.29 mmol), and *t*-BuOH/water (10 mL). The reaction mixture was stirred overnight at 60°C. After completion, water (14 mL) was added dropwise. The obtained suspension was cooled to RT and stirred for 1 hour at RT. The precipitate was filtered off, washed with DCM (3 mL), and dried. Compound **4** was obtained as a yellowish solid (133 mg, 65.2%, mp >200°C). ^1^H-NMR (400 MHz, DMSO-*d_6_*) δ 9.02 (1H, s, H-triaz.), 7.80 (4H, m, H-4,5,6,7), 7.43 (3H, m, H-6',6”,8'), 6.64 (2H, s, CH_2_), 5.59 (1H, s, OH) ppm [MH^+^ = 352.^3^].

##### 3.1.3.3 N-((1-(4-fluorophenyl)-1H-1,2,3-triazol-4-yl) methyl)benzo[d]oxazol-2-amine 5

Compound **5** was prepared according to the general procedure using compound **2** (200 mg, 1.16 mmol), 1-azido-4-fluorobenzene (2 mL, 1.05 mmol), CuSO_4_ x 5 H_2_O (173 µL, 0.12 mmol), sodium ascorbate (115 mg, 0.58 mmol), and *t*-BuOH/water (20 mL). The reaction mixture was stirred for 45 minutes at 70°C. After completion, the reaction mixture was evaporated to dryness. DCM (20 mL) and water (20 mL) were added, and the pH was adjusted to 2 with 2M HCl. After separation of the layers, the pH of the aqueous layer was adjusted to 8.7 with 1 M NaOH, resulting in precipitation. The suspension was stirred for 2 hours at RT. The precipitate was filtered off, washed with water (2 x 5 mL), and dried. Compound **5** was obtained as a beige solid (63 mg, 17.5%, mp = 137−139°C). ^1^H-NMR (400 MHz, DMSO-*d_6_*) δ 8.79 (1H, s, H-triaz.), 7.91 (2H, m, H-6',6''), 7.41 (2H, t, *J* = 8.76 Hz, H-7',7''), 7.13 (2H, m, H-4,7), 7.06 (1H, t, *J* = 7.63 Hz, H-5), 6.94 (1H, t, *J* = 7.70 Hz, H-6), 5.13 (2H, s, CH_2_) ppm. ^13^C-NMR (100 MHz, DMSO-*d_6_*) δ 162.58 (d, *J* = 246.36 Hz, C-8'), 162.45 (C-2), 143.56 (C-7a), 142.57 (C-3a), 132.84 (C-5'), 132.33 (C-3') 122.87 (C-5), 122.21 (d, *J* = 8.83 Hz, C-6',6''), 121.63 (C-4'), 120.34 (C-6), 116.13 (d, *J* = 23.41 Hz, C-7',7''), 107.85 (C-4), 107.72 (C-7), 36.98 (C-2') ppm [MH^+^ = 310.^2^].

##### 3.1.3.4 N-((1-(4-chlorophenyl)-1H-1,2,3-triazol-4-yl) methyl)benzo[d]oxazol-2-amine 6

Compound **6** was prepared according to the general procedure using compound **2** (200 mg, 1.16 mmol), 1-azido-4-chlorobenzene (2 mL, 1.05 mmol), CuSO_4_ x 5 H_2_O (173 µL, 0.12 mmol), sodium ascorbate (115 mg, 0.58 mmol), and *t*-BuOH/water (20 mL). The reaction mixture was stirred for 45 minutes at 70°C. After completion, the reaction mixture was evaporated to dryness. DCM (20 mL) and water (20 mL) were added, and the pH was adjusted to 2 with 2M HCl, resulting in precipitation. The suspension was stirred for 1 hour at RT. The precipitate was washed with water (2 mL) and dried. Compound **6** was obtained as a beige solid (51 mg, 12.5%, mp = 211-213°C). ^1^H-NMR (400 MHz, DMSO-*d_6_*) δ 8.94 (1H, s, H-triaz.), 7.89 (2H, d, *J* = 8.85 Hz, H-6',6''), 7.67 (2H, d, *J* = 8.85 Hz, H-7,7''), 7.60 (1H, d, *J* = 8,17 Hz, H-4), 7.54 (1H, d, *J* = 7.72 Hz, H-7), 7.36 (1H, t, *J* = 7.95 Hz, H-5), 7.28 (1H, t, *J* = 7.95 Hz, H-6), 5.50 (2H, s, CH_2_) ppm. ^13^C-NMR (100 MHz, DMSO-*d_6_*) δ 157.63 (C-2), 143.91 (C-7a), 141.39 (C-3a), 135.19 (C-5'), 133.17 (C-8'), 130.50 (C-3'), 129.92 (C-7',7''), 125.40 (C-5), 123.94 (C-6), 122.48 (C-4'), 121.83 (C-6',6''), 110.80 (C-4), 110.49 (C-7), 38.16 (C-2') ppm [MH^+^ = 326.^2^].

##### 3.1.3.5 4-(4-((benzo[d]oxazol-2-ylamino)methyl)-1H -1,2,3-triazol-1-yl)benzoic Acid 7

Compound **7** was prepared according to the general procedure using compound **2** (100 mg, 0.58 mmol), 4-azidobenzoic acid (86 mg, 0.52 mmol), CuSO_4_ x 5 H_2_O (86.5 µL, 0.058 mmol), sodium ascorbate (58 mg, 0.29 mmol), and *t*-BuOH/water (10 mL). The reaction mixture was stirred for 1 hour at 70°C. After completion, it was cooled to RT and stirred for an additional 1 hour. The precipitate was filtered off, washed with a 1:1 *t-*BuOH/water mixture (2 × 2 mL), and dried. Compound **7** was obtained as a yellowish solid (89 mg, 45.6%, mp > 250°C). ^1^H-NMR (400 MHz, DMSO-*d_6_*+trifluoroacetic acid) δ 8.91 (1H, s, H-triaz.), 8.07 (2H, d, *J* = 8.48 Hz, H-7',7''), 7.88 (2H, d, *J* = 8.48 Hz, H-6',6''), 7.62 (1H, d, *J* = 7.72 Hz, H-4), 7.55 (1H, d, *J* = 8.14 Hz, H-7), 7.34 (1H, t, *J* = 7.45 Hz, H-5), 7.28 (1H, t, *J* = 7.63 Hz, H-6), 5.55 (2H, s, CH_2_) ppm [MH^+^ = 336.^3^].

##### 3.1.3.6 N-((1-(4-bromophenyl)-1H-1,2,3-triazol-4-yl) methyl)benzo[d]oxazol-2-amine 8

Compound **8** was prepared according to the general procedure using compound **2** (200 mg, 1.16 mmol), 1-azido-4-bromobenzene (2 mL, 1.05 mmol), CuSO_4_ x 5 H_2_O (173 µL, 0.12 mmol), sodium ascorbate (115 mg, 0.58 mmol), and *t*-BuOH/water (20 mL). The reaction mixture was stirred for 1 hour at 70°C. After completion, it was evaporated to dryness. DCM (20 mL) and water (20 mL) were added, and the pH was adjusted to 9 with 2.5 M NaOH. After separation of layers, the pH of the organic layer was adjusted to 2 with 2 M HCl, resulting in precipitation. The suspension was stirred for 1 hour at RT. The precipitate was filtered off, washed with DCM (2 mL), and dried. Compound **8** was obtained as a yellowish solid (78 mg, 18.2%, mp > 250°C). ^1^H-NMR (400 MHz, DMSO-*d_6_* + trifluoroacetic acid) δ 9.03 (1H, s, H-triaz.), 7.81 (4H, s, H-6',6'',7',7''), 7.72 (1H, dd, *J* = 8.04, 0.99 Hz, H-4), 7.66 (1H, dd, *J* = 7.94, 1.03 Hz, H-7), 7.44 (1H, td, *J* = 7.85, 1.24 Hz, H-5), 7.38 (1H, td, *J* = 7.98, 1.32 Hz, H-6), 5.69 (2H, s, CH_2_) ppm. ^13^C-NMR (100 MHz, DMSO-*d_6_* + trifluoroacetic acid) δ 158.27 (C-2), 143.98 (C-7a), 140.98 (C-3a), 135.51 (C-5'), 132.89 (C-7',7''), 129.87 (C-3'), 125.95 (C-5), 124.82 (C-6), 122.74 (C-4'), 122.07 (C-6',6''), 121.64 (C-8'), 111.71 (C-4), 111.10 (C-7), 38.49 (C-2') ppm [MH^+^ = 370.2, 372.2- isotopes of Br].

##### 3.1.3.7 N-((1-(4-methoxyphenyl)-1H-1,2,3-triazol- 4-yl)methyl)benzo[d]oxazol-2-amine 9

Compound **9** was prepared according to the general procedure using compound **2** (200 mg, 1.16 mmol), 1-azido-4-methoxybenzene (2 mL, 1.05 mmol), CuSO_4_ x 5 H_2_O (173 µL, 0.12 mmol), sodium ascorbate (115 mg, 0.58 mmol), and *t*-BuOH/water (20 mL). The reaction mixture was stirred for 2 hours at 70°C. After completion, the reaction mixture was filtered off, and the mother liquor was evaporated to dryness. DCM (20 mL) and water (20 mL) were added, and the pH was set to 10 with 2.5 M NaOH. The layers were separated, and the pH of the organic layer was adjusted to 2 with 2 M HCl, resulting in precipitation. The suspension was stirred for 1 hour at RT. The precipitate was filtered off, washed with DCM (2 mL), and dried. Compound **9** was obtained as a yellowish solid (78 mg, 18.2%, mp > 200°C). δ 8.93 ^1^H-NMR (400MHZ, DMSO-*d*_6_ (1H, s, H-triaz.), 7.72 (3H, m, H-Ph, H-4,6',6''), 7.67 (1H, dd, *J* = 7.99, 0.86 Hz, H-7), 7.44 (1H, td, *J* = 7.84, 1.11 Hz, H-5), 7.37 (1H, td, *J* = 7.97, 1.27 Hz, H-6), 7.12 (2H, m, H-7',7''), 5.71 (2H, s, CH_2_), 3.81 (3H, s, OCH_3_) ppm. ^13^C-NMR (100 MHz, DMSO-*d_6_* + trifluoroacetic acid) δ 159.43 (C-2), 158.22 (C-8'), 143.97 (C-7a), 140.50 (C-3a), 129.93 (C-3'), 129.73 (C-5'), 125.92 (C-5), 124.76 (C-6), 122.81 (C-4'), 121.86 (C-6',6''), 114.96 (C-7',7''), 111.77 (C-4), 111.06 (C-7), 55.60 (C-2'), 38.58 (C-9') ppm [MH^+^ = 322.^3^].

##### 3.1.3.8 N-((1-phenyl-1H-1,2,3-triazol-4-yl)methyl) benzo[d]oxazol-2-amine 10

Compound **10** was prepared according to the general procedure using compound **2** (200 mg, 1.16 mmol), azidobenzene (2 mL, 1.05 mmol), CuSO_4_ x 5 H_2_O (173 µL, 0.12 mmol), sodium ascorbate (115 mg, 0.58 mmol), and *t*-BuOH/water (20 mL). The reaction mixture was stirred for 4.5 hours at 70°C. After completion, the reaction mixture was filtered off, and the mother liquor was evaporated to dryness. DCM (20 mL) and water (20 mL) were added, and pH was adjusted to 9.5 with 2.5 M NaOH. Layers were separated, and the pH of the organic layer was adjusted to 2 with 2 M HCl, resulting in precipitation. The suspension was stirred for 3 hours at RT. The precipitate was filtered off, washed with DCM (2 mL), and dried. Compound **10** was obtained as a yellowish solid (43 mg, 14.1%, mp = 226−228°C). ^1^H-NMR (400 MHz, DMSO-*d_6_* + trifluoroacetic acid) δ 9.04 (1H, s, H-triaz.), 7.83 (2H, m, H-6',6''), 7.70 (2H, m, H-4,7), 7.60 (2H, m, H-7',7''), 7.50 (1H, m, H-8'), 7.44 (1H, td, *J* = 7.88, 1.11 Hz, H-5), 7.37 (1H, td, *J* = 7.96, 1.31 Hz, H-6), 5.73 (1H, s, CH_2_) ppm. ^13^C-NMR (100 MHz, DMSO-*d_6_* + trifluoroacetic acid) δ 158.25 (C-2) 143.96 (C-7a), 140.75 (C-3a), 136.34 (C-5',) 129.99 (C7',7''), 128.99 (C-8'), 125.92 (C-5), 124.77 (C-6), 122.80 (C-4'), 120.15 (C-6',6''), 111.75 (C.4), 111.06 (C-7), 38.53 (C-8') ppm [MH^+^ = 292.^3^].

##### 3.1.3.9 N-((1-((phenylthio)methyl)-1H-1,2,3-triazol- 4-yl)methyl)benzo[d]oxazol-2-amine 11

Compound **11** was prepared according to the general procedure using compound **2** (200 mg, 1.16 mmol), (azidomethyl)phenyl sulfide (173 mg, 1.05 mmol), CuSO_4_ x 5 H_2_O (173 µL, 0.12 mmol), sodium ascorbate (115 mg, 0.58 mmol), and *t*-BuOH/water (20 mL). The reaction mixture was stirred for 4.5 hours at 70°C. After completion, the reaction mixture was filtered off, and the mother liquor was evaporated to dryness. DCM (20 mL) and water (20 mL) were added, and the pH was adjusted to 10 with 2.5 M NaOH. After separation, the organic layer was evaporated to dryness. The residue was dissolved in MeOH (2 mL), leading to precipitation. The suspension was stirred for 2 hours at RT. The precipitate was filtered off, washed with MeOH (0.5 mL), and dried. Compound **11** was obtained as a white solid (13 mg, 3.3%, mp 110−112°C). ^1^H-NMR (400 MHz, DMSO-*d_6_*) δ 8.11 (1H, s, H-triaz.), 7.23 (9H, m, H-4,5,6,7,7',7”,8',8”,9'), 5.89 (2H, s, H-5'), 5.08 (2H, s, H-2') ppm [MH^+^ = 339.^3^].

##### 3.1.3.10 2-(4-((benzo[d]oxazol-2-ylamino)methyl) -1H-1,2,3-triazol-1-yl)acetate 12

Compound **12** was prepared according to the general procedure using compound **2** (200 mg, 1.16 mmol), ethyl azidoacetate (150 mg, 1.16 mmol), CuSO_4_ x 5 H_2_O (173 µL, 0.12 mmol), sodium ascorbate (115 mg, 0.58 mmol), and *t*-BuOH/water (20 mL). The reaction mixture was stirred for 15 minutes at 70°C. After completion, EtOAc (30 mL) was added. Layers were separated, and the aqueous layer was evaporated to dryness. The obtained red solid was recrystallized from 2-PrOH (1 mL). Compound **12** was obtained as a reddish solid (40 mg, 11.4%, mp = 161−163°C). ^1^H-NMR (400 MHz, D_2_O) δ 8.20 (1H, s, H-triaz.), 7.47 (1H, d *J* = 7.28 Hz, H-5), 7.35 (3H, m, H-4,6,7), 5.45 (2H, s, H-5'), 5.30 (2H, s, H-2'), 4.15 (2H, q, *J* = 7.12 Hz, H-7'), 1.14 (3H, t, *J* = 7.20 Hz, H-8') ppm. ^13^C-NMR (100 MHz, D_2_O) δ 168.55 (C-2), 158.66 (C-6'), 144.18 (C-7a), 139.58 (C-3a), 129.16 (C-3'), 126.43 (C-4'), 126.25 (C-5), 125.51 (C-6), 11.29 (C-4), 111.20 (C-7), 63.36 (C-7'), 51.14 (C-5'), 38.42, (C-2'), 13.1 (C-8') ppm [MH^+^ = 302.^3^].

##### 3.1.3.11 N-((1-benzyl-1H-1,2,3-triazol-4-yl)methyl) benzo[d]oxazol-2-amine 13

Compound **13** was prepared according to the general procedure using compound **2** (100 mg, 0.58 mmol), benzyl azide (1 mL, 0.52 mmol), CuSO_4_ x 5 H_2_O (86 µL, 0.058 mmol), sodium ascorbate (58 mg, 0.29 mmol), and *t*-BuOH/water (10 mL). The reaction mixture was stirred for 20 minutes at 70°C. After completion, the obtained yellowish suspension was cooled to RT and stirred for 1 hour. The precipitate was filtered off, washed with a 1:1 *t-*BuOH/water mixture (2 × 1 mL), and dried. Compound **13** was obtained as a yellowish solid (22 mg, 9.6%, mp = 152−154°C). ^1^H-NMR (400 MHz, DMSO-*d_6_*) δ 8.30 (1H, s, H-triaz.), 7.68 (1H, d, *J* = 7.86 Hz, H-4), 7.58 (1H, d, *J* = 7.13 Hz, H-7), 7.34 (7H, m, H-5,6,7',7”,8',8”,9'), 5.58 (2H, s, H-5'), 5.43 (1H, s, H-2') ppm. ^13^C-NMR (100 MHz, DMSO-*d_6_* + trifluoroacetic acid) δ 157.70 (C-2), 143.36 (C-7a), 135.06 (C-3a), 129.32 (C-9'), 128.18 (C-8',8''), 127.65 (C-4'), 127.43 (C-7',7''), 125.37 (C-5), 124.29 (C-6), 123.98 (C-3'), 111.11 (C-4), 110.57 (C-7), 52.41 (C-5'), 37.92 (C-9') ppm [MH^+^ = 306.^3^].

##### 3.1.3.12 N-((1-(p-tolyl)-1H-1,2,3-triazol-4-yl)methyl) benzo[d]oxazol-2-amine 14

Compound **14** was prepared according to the general procedure using compound **2** (200 mg, 1.16 mmol), 4-azidotoluene (2 mL, 0.99 mmol), CuSO_4_ x 5 H_2_O (346 µL, 0.23 mmol), sodium ascorbate (115 mg, 0.58 mmol), and *t*-BuOH/water (10 mL). The reaction mixture was stirred for 1 hour at 70°C. After completion, the obtained suspension was cooled to RT and filtered off. The mother liquor was evaporated to dryness. EtOH (2 mL) was added, and the reaction mixture was stirred at reflux for 1 hour. After cooling to RT, the mixture was stirred for 1 hour, then cooled to 0°C and stirred for an additional 1 hour. The precipitate was filtered off, washed with EtOH (1 mL), and dried. Compound **14** was obtained as a white solid (32 mg, 9.3%, mp > 250°C). ^1^H-NMR (400 MHz, DMSO-*d_6_*) δ δ 8.87 (1H, s, H-triaz.), 7.72 (2H, d, *J* = 8.50 Hz, H-H-6',6''), 7.6 (1H, d, *J* = 7.58 Hz, H-4), 7.55 (1H, d, *J* = 7.88 Hz, H-7), 7.37 (3H, m, H-5,7',7''), 7.29 (1H, td, *J* = 7.80, 1.31 Hz, H-6), 5.49 (2H, s, CH_2_), 2.36 (3H, s, CH3) ppm. ^13^C-NMR (100 MHz, DMSO-*d_6_*) δ 157.58 (C-2), 143.92 (C-7a), 141.01 (C-3a), 138.60 (C-8''), 134.14 (C-5'), 130.44 (C-3'), 130.30 (C-6',6''), 125.44 (C-5), 124.02 (C-6), 122.25 (C-4'), 119.99 (C-7',7''), 110.89 (C-4), 110.55 (C-7), 38.24 (C-2'), 20.55 (C-9') ppm [MH^+^ = 306.^3^].

##### 3.1.3.13 N-((1-(3-(trifluoromethyl)phenyl)-1H-1,2,3-triazol-4-l)methyl)benzo[d]oxazol-2-amine 15

Compound **15** was prepared according to the general procedure using compound **2** (200 mg, 1.16 mmol), 1-azido-3-(trifluoromethyl)benzene (2 mL, 0.99 mmol), CuSO_4_ x 5 H_2_O (346 µL, 0.23 mmol), sodium ascorbate (115 mg, 0.58 mmol), and *t*-BuOH/water (10 mL). The reaction mixture was stirred for 10 minutes at 70°C. After completion, it was cooled to RT and stirred for 1 hour. The precipitate was filtered off, washed with a 1:1 t-BuOH/water mixture (2× 1 mL), and dried. Compound **15** was obtained as a yellow solid (113 mg, 27.1%, mp =171−173°C). ^1^H-NMR (400 MHz, DMSO-*d_6_*) δ 9.08 (1H, s, H-triaz.), 8.20 (2H, m, H-8',10'), 7.85 (2H, m, H- -6',7'), 7.73 (1H, d, *J* = 7.82 Hz, H-4), 7.65 (1H, d, *J* = 7.67 Hz, H-7), 7.41 (2H, m, H-5,6), 5.62 (2H, s, CH_2_) ppm. ^13^C-NMR (100 MHz, DMSO-*d_6_*) δ 143.55 (C2), 140.53 (C-7a), 136.39 (C-3a), 131.04 (C-6'), 130.33 (C-3'), 129.98 (C-9'), 129.36 (C-5'), 125.66 (C-8'), 125.16 (C-5), 124.51 (C-7'), 124.44 (C-11'), 122.58 (C-4'), 116.38 (C-10'), 111.29 (C-4), 110.73 (C-7), 38.07 (C-2') ppm [MH^+^ = 360.^3^].

#### 3.1.4 Synthesis of 2-mercaptobenzo[d]oxazole 16

2-aminophenol (20.0 g, 183.2 mmol) was dissolved in MeOH (200 mL), and a solution of KOH (13.4 mg, 238.2 mmol) in water (56 mL) and CS_2_ (16.5 mL, 274.8 mmol) was added dropwise. The reaction mixture was stirred for 5 hours at 65°C, then cooled to RT. Water (673 mL) was added dropwise, and the pH was adjusted to 7 with conc. HCl, resulting in precipitation. The suspension was stirred for 30 minutes at RT, cooled to 0-5°C, and stirred for an additional 1 hour. The resulting crystals were filtered off, washed with MeOH/water (1:10, 2 × 20 mL), and dried at 60°C under 10 mbar for 10 hours. Compound **16** was obtained as a beige solid (16.2 g, 58.5%). ^1^H-NMR (400 MHz, DMSO-*d_6_*) δ 13.83 (1H, bs, SH), 7.50 (1H, m, H-5), 7.27 (3H, m, H-4,6,7) ppm. ^13^C-NMR (100 MHz, DMSO-*d_6_*) δ 180.15 (C-2) 148.12 (C-7a), 131.22 (C-3a), 125.17 (C-5), 123.81 (C-6), 110.48 (C-4), 109.93 (C-7) ppm [MH^+^ = 152.^2^].

#### 3.1.5 Synthesis of 2-(prop-2-yn-1-ylthio)benzo[d]oxazole 17

Compound **16** (16.2 g, 107.2 mmol) was dissolved in acetone (162 mL), and an aqueous solution of K_2_CO_3_ (29.6 g, 214.3 mmol) was added. The reaction mixture was cooled to 0−5°C, and propargyl bromide (9.2 M solution in toluene, 12.8 mL, 117.9 mmol) was added dropwise. The reaction mixture was stirred for 30 minutes at RT, followed by the dropwise addition of water (540 mL), which led to precipitation. The suspension was stirred at RT for 30 minutes, cooled to 0−5°C, and stirred for an additional 1 hour. The resulting crystals were filtered off, washed with water (100 mL), and dried at 60°C under 10 mbar for 10 hours. Compound **16** was obtained as **a** brown solid (16.9 g, 83.3%, T.t. <75°C) [24]. ^1^H-NMR (400 MHz, DMSO-*d_6_*) δ 7.64 (2H, m, H-4,7), 7.34 (2H, m, H-5,6), 4.21 (2H, d, *J* = 2.59, CH_2_), 3.29 (1H, t, *J* = 2.62 Hz, CH) ppm. ^13^C-NMR (100 MHz, DMSO-*d_6_*) δ 162.72 (C-2), 151.37 (C-7a), 141.18 (C-3a), 124.75 (C-5), 124.50 (C-6), 118.47 (C-4), 110.29 (C-7), 74.57 (C-2'), 20.30 (C-3') ppm [MH^+^ = 190.^2^].

#### 3.1.6 General Procedure for the Synthesis of 1,2,3-triazole derivatives of 2-mercaptobenzoxazole 18−32

A mixture of compound **17** (1 eq.), the corresponding azide (1.1 eq.), CuSO_4_ x 5 H_2_O (0.1 eq., as a 0.67 M aqueous solution), sodium ascorbate (0.5 eq.), and *t*-BuOH/water (1:1) was stirred at 70°C. After completion of the reaction (monitored by UHPLC), the reaction mixture was evaporated to dryness. MeOH/water = 1/1 was added dropwise, or the residue was cooled to RT to induce precipitation. The obtained suspension was stirred for 1 hour at RT, and the precipitate was filtered off, washed with MeOH/water, and dried at 50°C under 10 mbar for 8 hours.

##### 3.1.6.1 2-(((1-(3-(trifluoromethyl)phenyl)-1H-1,2,3-triazol-4-yl)methyl)thio)benzo[d] oxazole 18

Compound **18** was prepared according to the general procedure using compound **17** (200 mg, 1.06 mmol), 1-azido-3-(trifluoromethyl)benzene (1.8 mL, 0.90 mmol), CuSO_4_ x 5 H_2_O (316 µL, 0.21 mmol), sodium ascorbate (105 mg, 0.53 mmol), and *t*-BuOH/water (8 mL). The reaction mixture was stirred for 20 minutes at 70°C. After completion, the reaction mixture was evaporated to dryness, and MeOH/water (1:2, 20 mL) was added. The obtained suspension was stirred for 1 hour at RT. The precipitate was filtered off, washed with MeOH/water (1:5, 2 × 1 mL), and dried. Compound **18** was obtained as a yellowish solid (93 mg, 23.4%, mp = 90−92°C). ^1^H-NMR (400 MHz, DMSO-*d_6_*) δ 9.02 (1H, s, H-triaz.), 8.24 (2H, m, H-6',10'), 7.82(2H, m, H-7',8'), 7.65 (2H, m, H-4,7), 7.33 (2H, m, H-5,6), 4.78 (2H, s, CH_2_) ppm. ^13^C-NMR (100 MHz, DMSO-*d_6_*) δ 163.37 (C-2), 151.44 (C-7a), 144.01 (C-3a), 141.24 (C-3'), 136.98 (C-5'), 131.28 (C-6'), 130.49 (q, *J* = 32.33 Hz, C-11'), 125.26 (C-5), 124.89 (C-6), 124.66 (C-8'), 124.37 (C-5), 124.04 (C-6), 122.44 (C-4'), 122.18 (C-9') 118.41 (C-4). 116.83 (C-10'), 110.30 (C-7), 26.32 (C-2') ppm [MH^+^ = 377.^3^].

##### 3.1.6.2 2-(((1-(p-tolyl)-1H-1,2,3-triazol-4-yl)methyl) thio)benzo[d]oxazole 19

Compound **19** was prepared according to the general procedure using compound **17** (200 mg, 1.06 mmol), 4-azidotoluene (1.8 mL, 0.90 mmol), CuSO_4_ x 5 H_2_O (632 µLµ, 0.42 mmol), sodium ascorbate (105 mg, 0.53 mmol), and *t*-BuOH/water (8 mL). The reaction mixture was stirred for 30 minutes at 70°C. After completion, the reaction mixture was cooled to RT and stirred for 1 hour. The precipitate was filtered off, washed with *t*-BuOH/water (1:1, 2 mL), suspended in MeOH (5 mL), and stirred at RT for 2 hours. Precipitation occurred in the mother liquor, which was filtered. The precipitate was washed with MeOH/water (1:3, 2 mL) and dried at 60°C under 10 mbar for 6 hours. Compound **19** was obtained as a white to pale yellow solid (53 mg, 15.5%, mp = 110−112°C). ^1^H-NMR (400 MHz, DMSO-*d_6_*) δ 8.78 (1H, s, H-triaz.), 7.74 (2H, m, H-6',6''), 7.67 (2H, m, H-4,7.), 7.37 (2H, m, H-7',7'') 7.33 (2H, m, H-5,6), 4.76 (2H, s, CH_2_), 2.36 (3H, s, CH3) ppm. ^13^C-NMR (100 MHz, DMSO-*d_6_*) δ 163.45 (C-2), 151.38 (C-7a), 143.40 (C-3a), 141.2 (C-8'), 138.36 (C-5'), 134.(C-3'), 130.19 (C-6',6''), 124.64 (C-5), 124.33 (C-6), 121.92 (C-4'), 119.97 (C-7',7''), 118.39 (C-4), 110.29 (C-7), 26.37 (C-7'), 20.53 (C-9') ppm [MH^+^ = 323.^3^].

##### 3.1.6.3 2-(((1-benzyl-1H-1,2,3-triazol-4-yl)methyl) thio)benzo[d]oxazole 20

Compound **20** was prepared according to the general procedure using compound **17** (200 mg, 1.06 mmol), benzyl azide (2 mL, 1.0 mmol), CuSO_4_ x 5 H_2_O (316 µL, 0.21 mmol), sodium ascorbate (105 mg, 0.53 mmol), and *t*-BuOH/water (4 mL). The reaction mixture was stirred for 20 min at 70°C. After completion, the reaction mixture was evaporated to dryness, and the residue was suspended in MeOH/water (1:1, 4 mL). The mixture was heated at 50°C for 30 minutes, cooled to 0°C, and stirred for 1 hour. The precipitate was filtered off, washed with MeOH/water (1:2, 2 mL), and dried. Compound **20** was obtained as a yellow solid (50 mg, 15.5%, mp = 115−117°C). ^1^H-NMR (400 MHz, DMSO-*d_6_*) δ 8.22 (1H, s, H-triaz.), 7.66 (2H, m, H-4,7), 7.30 (7H, m, H-5,6,7',7”,8',8”,9'), 5.56 (2H, s, CH_2_'), 4.68 (2H, s, CH_2_) ppm. 13C- NMR (100 MHz, DMSO-*d_6_*) δ 163.77 (C-2), 151.23 (C-7a), 142.79 (C-3a), 141.07 (C-6'), 135.84 (C-3'), 128.72 (C-8',8''), 128.11 (C-9'), 127.86 (C-7',7''), 124.67 (C-5), 124.47 (C-6), 124.18 (C-4'), 118.26 (C-4), 110.25 (C-7), 52.87 (C-5'), 26.36 (C-2') ppm [MH^+^ = 323.^3^].

##### 3.1.6.4 Ethyl 2-(4-((benzo[d]oxazol-2-ylthio)methyl) -1H-1,2,3-triazol-1-yl)acetate 21

Compound **21** was prepared according to the general procedure using compound **17** (200 mg, 1.06 mmol), ethyl azidoacetate (123mg, 0.95 mmol), CuSO_4_ x 5 H_2_O (316 µL, 0.21 mmol), sodium ascorbate (105 mg, 0.53 mmol), and *t*-BuOH/water (8 mL). The reaction mixture was stirred for 20 minutes at 70°C. After completion, the reaction mixture was cooled to RT and filtered. The mother liquor was evaporated to dryness, and the residue was suspended in MeOH/water (2:1, 1.5 mL), stirred at RT for 1 hour at RT. The precipitate was filtered off, washed with MeOH/water (1:2, 1 mL), and dried. Compound **21** was obtained as a yellowish solid (24 mg, 7.1%, mp = 95−97°C). ^1^H-NMR (400 MHz, DMSO-*d_6_* + trifluoroacetic acid) δ 8.14 (1H, s, H-triaz.), 7.66 (2H, m, H-4,7), 7.33 (2H, m, H-5,6), 5.34 (2H, s, H-5'), 4.69 (2H, s, H-2'), 4.13 (2H, q, *J* = 6.99 Hz, H-7'), 1.17 (3H, t, *J* = 6.95 Hz, CH3) ppm. ^13^C-NMR (100 MHz, DMSO-*d_6_* + trifluoroacetic acid) δ 167.32 (C-6'), 160.35 (C-2) 141.69 (C-7a), 137.86 (C-3a), 124.67 (C-5), 124.37 (C-6), 118.54 (C-4), 110.30 (C-7), 84.43 (C-7'), 61.64 (C-5'), 50.74 (C-2'), 13.76 (C-8') ppm [MH^+^ = 319.^3^].

##### 3.1.6.5 2-(((1-((phenylthio)methyl)-1H-1,2,3-triazol-4-yl)methyl)thio)benzo[d]oxazole 22

Compound **22** was prepared according to the general procedure using compound **17** (300 mg, 1.59 mmol), (azidomethyl)phenyl sulfide (234 mg, 1.43 mmol), CuSO_4_ x 5 H_2_O (474 µL, 0.32 mmol), sodium ascorbate (158 mg, 0.80 mmol), and *t*-BuOH/water (25 mL). The reaction mixture was stirred for 30 minutes at 70°C. After completion, the reaction mixture was cooled to RT and filtered. The mother liquor was evaporated to dryness. Compound **22** was obtained as a yellow oil (350 mg, 62.3%). ^1^H-NMR (400 MHz, DMSO-*d_6_*) δ 8.07 (1H, s, H-triaz.), 7.61 (2H, m, H-4,7) 7.26 (7H, m, H-H,5,6,7',7”,8',8”,9'), 5.9 (2H, s, H-5'), 4.64 (2H, s, H-2') ppm. ^13^C-NMR (100 MHz, DMSO-*d_6_*) δ 163.79 (C-2), 151.20 (C-7a), 143.10 (C-3a), 141.02 (C-6'), 132.09 (C-3'), 130.75 (C7'.7''), 129.14 (C8',8''), 127.74 (C-9'), 124.69 (C-5), 124.49 (C-6), 123.72 (C-4'), 118.27 (C-4), 110.26 (C-7), 51.90 (C-5'), 26.19 (C-2') ppm [MH^+^ = 355.^3^].

##### 3.1.6.6 4-((4-((benzo[d]oxazol-2-ylthio)methyl)-1H-1, 2,3-triazol-1-yl)methyl)benzonitrile 23

Compound **23** was prepared according to the general procedure using compound **17** (200 mg, 1.06 mmol), 4-(azidomethyl)benzonitrile (1.9 mL, 0.95 mmol), CuSO_4_ x 5 H_2_O (316 µL, 0.21 mmol), sodium ascorbate (105 mg, 0.53 mmol), and *t*-BuOH/water (8 mL). The reaction mixture was stirred for 30 minutes at 70°C. After completion, the reaction mixture was cooled to RT and filtered. The mother liquor was evaporated to dryness. Compound **23** was obtained as a green solid (300 mg, 90.9%, mp = 134−136°C). ^1^H-NMR (400 MHz, DMSO-*d_6_*) δ 8.28 (1H, s, H-triaz.), 7.76 (2H, d, *J* = 8.06 Hz, H-8',8''), 7.58 (2H, m, H-4,7) 7.38 (2H, d, *J* = 8.12 Hz, H-7',7'') 7.30 (2H, m, H-5,6) 5.69 (2H, s, H-5'), 4.69 (2H, s, H-2') ppm [MH^+^ = 348.^3^].

##### 3.1.6.7 4-(4-((benzo[d]oxazol-2-ylthio)methyl)-1H-1, 2,3-triazol-1-yl)benzoic acid 24

Compound **24** was prepared according to the general procedure using compound **17** (300 mg, 1.59 mmol), 4-azidobenzoic acid (261 mg, 1.60 mmol), CuSO_4_ x 5 H_2_O (474 µL, 0.32 mmol), sodium ascorbate (158 mg, 0.80 mmol), and *t*-BuOH/water (12 mL). The reaction mixture was stirred for 30 minutes at 70°C. After completion, the reaction mixture was cooled to RT and stirred for 1 hour. The precipitate was filtered off, washed with *t*-BuOH/water (1:1, 2 mL), and dried. Compound **24** was obtained as a yellow solid (582 mg, 94.6%, mp = 242−244°C). ^1^H-NMR (400 MHz, DMSO-*d_6_*) δ 8.93 (1H, s, H-triaz.), 8.05 (4H, bs, H-6',6”,7',7''), 7.65 (2H, m, H- 4,7), 7.31 (2H, m, H-5,6), 4.79 (2H, s, CH_2_) ppm. ^13^C-NMR (100 MHz, DMSO-*d_6_*) δ 163.62 (C-2), 151.29 (C-7a), 144.16 (C-3a), 141.10 (C-5'), 139.17 (C-3'), 124.70 (C-7',7''), 124.45 (C,6',6''), 122.31 (C-5), 120.11 (C-4'), 118.38 (C-4), 110.31 (C-7), 26.49 (C-2') ppm [MH^+^ = 353.^3^].

##### 3.1.6.8 2-(((1-((trimethylsilyl)methyl)-1H-1,2,3-triazol-4-yl)methyl)thio)benzo[d]oxazole 25

Compound **25** was prepared according to the general procedure using compound **17** (200 mg, 1.06 mmol), (azidomethyl)trimethylsilane (137 mg, 1.06 mmol), CuSO_4_ x 5 H_2_O (316 µLl, 0.21 mmol), sodium ascorbate (105 mg, 0.53 mmol), and *t*-BuOH/water (8 mL). The reaction mixture was stirred for 20 minutes at 70°C. After completion, the reaction mixture was evaporated to dryness. DCM (5 mL) and water (5 mL) were added to the evaporated residue. Layers were separated, and the organic layer was washed with water (5 mL) and evaporated to dryness. Compound **25** was obtained as a brown oil (180 mg, 53.5%). ^1^H-NMR (400 MHz, DMSO-*d_6_*) δ 7.95 (1H, s, H-triaz.), 7.64 (2H, s, H-4,7), 7.32 (2H, s, H-5,6) 4.64 (2H, s, H-2'), 3.96 (2H, s, H-5'), 0.00 (9H, s, (CH_3_)_3_) ppm. ^13^C-NMR (100 MHz, DMSO-*d_6_*) δ 163.58 (C-2), 151.25 (C-7a), 141.18 (C-3a), 124.61 (C-5), 124.30 (C-6), 118.32 (C-4), 110.24 (C-7), 40.90 (C-5'), 26.51 (C-2'), -2.74 (C-6',6”,6”') ppm [MH^+^ = 319.^4^].

##### 3.1.6.9 2-(4-((benzo[d]oxazol-2-ylthio)methyl)-1H-1, 2,3-triazol-1-yl)acetic acid 26

Compound **26** was prepared according to the general procedure using compound **17** (200 mg, 1.06 mmol), azidoacetic acid (107 mg, 1.06 mmol), CuSO_4_ x 5 H_2_O (316 µL, 0.21 mmol), sodium ascorbate (105 mg, 0.53 mmol), and *t*-BuOH/water (6 mL). The reaction mixture was stirred for 20 minutes at 70°C. After completion, the reaction mixture was added dropwise to water (25 mL). The obtained suspension was stirred for 1 hour at RT. Precipitate was filtered off, washed with *t*-BuOH/water (1:1, 2 mL), and dried. Compound **26** was obtained as a beige solid (196 mg, 63.9%, mp = 133−135°C). ^1^H-NMR (400 MHz, DMSO-*d_6_* + trifluoroacetic acid) δ 8.16 (1H, s, H-triaz.), 7.65 (2H, m, H-4,7), 7.32 (2H, m, H-5,6), 5.24 (2H, s, H-5'), 4.69 (2H, s, H-2') ppm. ^13^C-NMR (100 MHz, DMSO-*d_6_* + trifluoroacetic acid) δ 168.35 (C-6'), 163.67 (C-2), 151.38 (C-7a), 141.21 (C-3a), 125.49 (C-4'), 124.54 (C-5), 124.26 (C-6), 118.30 (C-4), 110.16 (C-7), 50.46 (C-5'), 26.35 (C-2') ppm [MH^+^ = 291.^3^].

##### 3.1.6.10 4-(4-((benzo[d]oxazol-2-ylthio)methyl)-1H -1,2,3-triazol-1-yl)-2,3,5,6-tetrafluorobenzoic acid 27

Compound **27** was prepared according to the general procedure using compound **17** (200 mg, 1.06 mmol), 4-azido-2,3,5,6-tetrafluorobenzoic acid (249 mg, 1.06 mmol), CuSO_4_ x 5 H_2_O (316 µL, 0.21 mmol), sodium ascorbate (105 mg, 0.53 mmol), and *t*-BuOH/water (12 mL). The reaction mixture was stirred for 1.5 hours at 70°C. After completion, the reaction mixture was cooled to RT and stirred for 1 hour. The precipitate was filtered off, washed with *t*-BuOH/water (1:1, 2 mL), and dried. Compound **27** was obtained as a yellowish solid (196 mg, 29.0%, mp = 131−133°C). ^1^H-NMR (400 MHz, DMSO-*d_6_* + trifluoroacetic acid) δ 8.67 (1H, s, H-triaz.), 7.61 (2H, s, H-4,7), 7.29 (2H, s, H-5,6), 4.81 (2H, s, CH_2_) ppm [MH^+^ = 425.^3^].

##### 3.1.6.11 3-(4-((benzo[d]oxazol-2-ylthio)methyl)-1H -1,2,3-triazol-1-yl)propyl) ethanethioate *28*

Compound **28** was prepared according to the general procedure using compound **17** (200 mg, 1.06 mmol), *S*-(3-azidopropyl)thioacetate (177 mg, 1.11 mmol), CuSO_4_ x 5 H_2_O (316 µL, 0.21 mmol), sodium ascorbate (105 mg, 0.53 mmol), and *t*-BuOH/water (8 mL). The reaction mixture was stirred for 20 minutes at 70°C. After completion, the reaction mixture was evaporated to dryness. DCM (5 mL) and water (5 mL) were added to the evaporated residue. Layers were separated, and the organic layer was washed with water (5 mL) and evaporated to dryness. Compound **28** was obtained as a yellow oil (150 mg, 40.8%). ^1^H-NMR (400 MHz, DMSO-*d_6_*) δ 8.16 (1H, s, H-triaz.), 7.65 (2H, m, H-4,7), 7.34 (2H, m, H-5,6), 4.67 (1H, s, H-2'), 4.37 (2H, t, *J* = 6.92 Hz, H-5'), 2.76 (2H, t, *J* = 7.06 Hz, H-7'), 2.29 (3H, s, H-9'), 2.02 (2H, q, *J* = 7.10, H-6') ppm. ^13^C-NMR (100 MHz, DMSO-*d_6_*) δ 195.07 (C-8'), 163.55 (C-2), 151.40 (C-7a), 142.32 (C-3a), 141.28 (C-3'), 124.65 (C-5), 124.34 (C-6), 123.91 (C-4'), 118.36 (C-4), 110.25 (C-7), 48.27 (C-5'), 30.50 (C-9'), 29.72 (C-7'), 26.47 (C-2'), 25.35 (C-6') ppm [MH^+^ = 349.^3^].

##### 3.1.6.12 4-(4-((benzo[d]oxazol-2-ylthio)methyl)-1H -1,2,3-triazol-1-yl)-2H-chromen-2-one 29

Compound **29** was prepared according to the general procedure using compound **17** (53 mg, 0.28 mmol), 4-azidocoumarin (53 mg, 0.28 mmol), CuSO_4_ x 5 H_2_O (80 µL, 0.05 mmol), sodium ascorbate (27 mg, 0.14 mmol), and *t*-BuOH/water (6 mL). The reaction mixture was stirred for 1 hour at 70°C. After completion, the reaction mixture was cooled to RT and stirred for 1h. The precipitate was filtered off, washed with *t*-BuOH/water (1:1, 2 mL), and dried. Compound **29** was obtained as a yellow solid (40 mg, 38.0%, mp = 151−153°C). ^1^H-NMR (400 MHz, DMSO-*d_6_*) δ 8.82 (1H, s, H-triaz.), 7.71 (4H, m, H-4,7,7',9'), 7.55 (1H, d, *J* = 8.39 Hz, H-8'), 7.40-7.31 (3H, m, H-5,6,10'), 6.92 (1H, s, H-13'), 4.82 (2H, s, H-2') ppm. ^13^C-NMR (100 MHz, DMSO-*d_6_*) δ 163.33 (C-2), 159.37 (C-12'), 153.59 (C-11'), 151.41 (C-7a), 145.75 (C-5'), 143.50 (C-3a), 141.20 (C-3'), 133.43 (C-9'), 125.71 (C-4'), 125.27 (C-8'), 124.89 (C-7'), 124.65 (C-5), 124.39 (C-6), 118.42 (C-4), 117.16 (C-10'), 110.72 (C-7), 110.32 (C-13'), 26.05 (C-2') ppm [MH^+^ = 377.^3^].

##### 3.1.6.13 2-(((1-(2-fluorobenzyl)-1H-1,2,3-triazol-4- yl)methyl)thio)benzo[d]oxazole 30

Compound **30** was prepared according to the general procedure using compound **17** (200 mg, 1.06 mmol), 1-azidomethyl-2-fluorobenzene (2.2 mL, 1.10 mmol), CuSO_4_ x 5 H_2_O (316 µL, 0.21 mmol), sodium ascorbate (105 mg, 0.53 mmol), and *t*-BuOH/water (8 mL). The reaction mixture was stirred for 1 hour at 70°C. After completion, the reaction mixture was cooled to RT, and the layers were separated. The organic layer was washed with water (2 × 5 mL) and evaporated to dryness. MeOH (4 mL) was added to the residue after evaporation. The reaction mixture was heated to 60°C, and water (4 mL) was added dropwise. The obtained suspension was stirred at 60°C for 30 minutes, cooled to RT, and stirred for 1 hour. The precipitate was filtered off, washed with MeOH/water (1:5, 3 mL) and water (3 mL), and dried. Compound **30** was obtained as a brown solid (111 mg, 30.9%, mp = 94−96°C). ^1^H-NMR (400 MHz, DMSO-*d_6_*) δ 8.20 (1H, s, H-triaz.), 7.64 (2H, m, H-4,7), 7.28 (6H, m, H-5,6,7',8',9',10'), 5.63 (2H, s, H-5'), 4.68 (2H, s, H-2') ppm. ^13^C-NMR (100 MHz, DMSO-*d_6_*) δ 163.64 (C-2), 159.97 (d, *J* = 246.37 Hz, C-11'), 151.24 (C-7a), 141.10 (C-3a), 130.67 (C-7'), 130.58 (C-8'), 124.76 (C-5), 124.63 (C-6), 124.40 (C-9'), 124.30 (C-4'), 122.74 (C-6'), 118.28 (C-4), 115.52 (d, *J* = 20.64 Hz, C-10'), 110.23 (C-7), 46.95 (C-5'), 26.33 (C-2') ppm [MH^+^ = 341.^3^].

##### 3.1.6.14 2-(((1-(2-chlorobenzyl)-1H-1,2,3-triazol-4- yl)methyl)thio)benzo[d]oxazole 31

Compound **31** was prepared according to the general procedure using compound **17** (200 mg, 1.06 mmol), 1-azidomethyl-2-chlorobenzene (2.2 mL, 1.10 mmol), CuSO_4_ x 5 H_2_O (316 µL, 0.21 mmol), sodium ascorbate (105 mg, 0.53 mmol), and *t*-BuOH/water (8 mL). The reaction mixture was stirred for 30 minutes at 70°C. After completion, the reaction mixture was cooled to RT, and the layers were separated. The organic layer was evaporated to dryness. DCM (5 mL) and water (5 mL) were added to the evaporated residue. The layers were separated, and the organic layer was washed with water (3 mL) and then evaporated to dryness. Compound **31** was obtained as a brownish solid (103 mg, 27.4%, mp = 106−108°C). ^1^H-NMR (400 MHz, DMSO-*d_6_*) δ 8.18 (1H, s, H-triaz.), 7.64 (2H, m, H-4,7), 7.47 (1H, dd, *J =* 7.96, 1.11 Hz, H- 10'), 7.33 (4H, m, H-5,6,6',8'), 7,15 (1H, dd, *J* = 7.78, 1.49 Hz, H-7'), 5.68 (2H, s, H-5'), 4.68 (2H, s, H-2') ppm. ^13^C-NMR (100 MHz, DMSO-*d_6_*) δ 163.48 (C-2), 151.29 (C-7a), 142.49 (C-6'), 141.19 (C-3a), 133.18 (C-11'), 132.54 (C-3'), 130.32 (C-7'), 130.18 (C-10'), 129.57 (C-9'), 127.64 (C-8'), 124.62 (C-5), 124.49 (C-4'), 124.36 (C-6', 118.33 (C-4), 110.23 (C-7), 50.59 (C-5), 26.35 (C-2') ppm [MH^+^ = 357.^3^].

##### 3.1.6.15 (4-((benzo[d]oxazol-2-ylthio)methyl)-1H-1, 2,3-triazol-1-yl)methyl pivalate 32

Compound **32** was prepared according to the general procedure using compound **17** (200 mg, 1.06 mmol), azidomethyl pivalate (171 mg, 1.09 mmol), CuSO_4_ x 5 H_2_O (316 µL, 0.21 mmol), sodium ascorbate (105 mg, 0.53 mmol), and *t*-BuOH/water (8 mL). The reaction mixture was stirred for 20 minutes at 70°C. After completion, the reaction mixture was evaporated to dryness. DCM (5 mL) and water (5 mL) were added to the evaporated residue. The layers were separated, and the organic layer was washed with water (3 mL) and then evaporated to dryness. Compound **32** was obtained as an orange oil (200 mg, 54.7%). ^1^H-NMR (400 MHz, DMSO-*d_6_*) δ 8.24 (1H, s, H-triaz.), 7.64 (2H, m, H-4,7), 7.33 (2H, m, H-5,6), 6.26 (2H, s, H-5'), 4.69 (2H, s, H-2'), 1.02 (9H, s, (CH_3_)_3_) ppm. ^13^C-NMR (100 MHz, DMSO-*d_6_*) δ 176.37 (C-6'), 163.41 (C-2), 151.33 (C-7a), 143.03 (C-3a), 141.18 (C-3'), 125.02 (C-4'), 124.66 (C-5), 124.40 (C-6), 118.37 (C-4), 110.27 (C-7), 70.05 (C-5'), 38.12 (C-7'), 26.37 (C-8',8”,8”'), 26.14 (C-2') ppm [MH^+^ = 347.^3^].

### 3.2 Computational Methods

#### 3.2.1 Calculation of ADME Properties

Free access to a comprehensive set of predictive models designed to assess physicochemical properties, drug-likeness, and suitability for medicinal chemistry based on molecular structure input is provided by the SwissADME (SwissDrugDesign) web tool (http://www.swissadme.ch) [62].

#### 3.2.2 Molecular Docking

Molecular docking studies were conducted on 1,2,3-triazole derivatives of benzoxazole with antiproliferative activities, targeting two kinases: non-receptor protein-tyrosine kinase (c-Src) and TAO2 kinase. The crystal structures of c-Src complexed with ligand DSA (PDB ID: 3G6H) and TAO2 kinase bound with inhibitor staurosporine (PDB ID: 2GCD) were obtained from the Protein Data Bank. The protein structures were prepared for molecular docking using BIOVIA Discovery Studio Visualizer 4.5 (Dassault Systèmes, France). The 3D structures of the ligands were optimized using Spartan’08 (Wavefunction, Inc.; Irvine, CA, USA, 2009) with the molecular mechanics force field (MM+) [63] followed by refinement *via* the semiempirical AM1 method [64]. Molecular docking of 12 optimized compounds was performed utilizing the iGEMDOCK (BioXGEM, Taiwan) with a generic evolutionary method (GA). The GA parameters were set as follows: a population size of 200, 70 generations, 3 poses, and a binding site radius of 8 Å. For molecular docking, interactions between ligands and proteins, specifically Electrostatic (Elec), Hydrogen-bonding (Hbond), and van der Waals (vdW) interactions, were considered as per the iGEMDOCK scoring function. Docked compounds were ranked based on the total energy of the predicted pose in the binding site, defined as: *E* (kcal mol^-1^) = vdW + Hbond + Elec; where the vdW is van der Waals energy, Hbond denotes hydrogen bonding energy, and Elec refers to electrostatic energy [65, 66].

### 3.3 Antiproliferative Evaluation

For proliferation assays, adherent cell lines LN-229, HCT-116, NCI-H460, and Capan-1 were plated in 384-well tissue culture plates (Greiner, Kremsmünster, Austria) at densities ranging from 500 to 1500 cells per well: 500 cells per well for Capan-1, 1000 for LN-229 and HCT-116, and 1500 for NCI-H460. Following overnight incubation, cells were exposed to seven concentrations of the test compounds in a 5-fold dilution series, spanning from 100 to 0.006 µM. For suspension cell lines HL-60, K-562, Z-138, and DND-41, seeding densities ranged from 2500 to 5500 cells per well: 2500 for HL-60, K-562, and Z-138, and 5500 for DND-41. These cells were plated in 384-well culture plates containing the test compounds at the same concentration range. All experimental conditions were incubated for 72 hours prior to assessing cell viability using the CellTiter 96^®^ Aqueous Non-Radioactive Cell Proliferation Assay (MTS/PMS), according to the manufacturer’s protocol. For the assay, 10 µL of the MTS/PMS solution was added to each well of a 384-well culture plate containing 50 µL of medium per well, resulting in final concentrations of 333 µg/mL MTS and 25 µM PMS. Absorbance was measured at 490 nm after a 3-hour incubation using a SpectraMax Plus 384 plate reader. The background absorbance, measured in wells without cells, was subtracted from the data. The resulting Optical Density (OD) values were utilized to calculate the 50% inhibitory concentrations (IC_50_). Each compound was evaluated in two independent experiments [67, 68].

## 4 RESULTS AND DISCUSSION

### 4.1 Chemistry

**Scheme 1:**
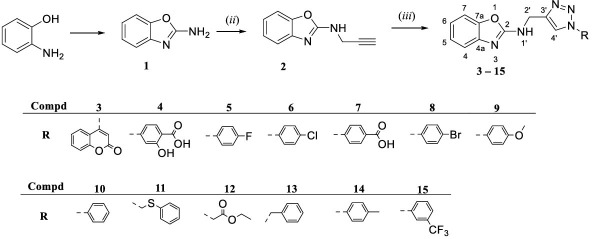
Reagents and conditions: (*i*) di(imidazole-1-yl)methaneimine, THF, reflux, (*ii*) prBr, DMF, (*iii*) RN_3_, CuSO_4_ x 5 H_2_O, sodium ascorbate, *t*-BuOH/H_2_O.

Novel 1,2,3-triazole derivatives of 2-aminobenzoxazole **3**−**15** and 2-mercaptobenzoxazole **18**−**32** were synthesized *via* a copper-catalyzed click reaction between propargylated benzoxazole derivatives and the corresponding azides. The 1,2,3-triazole derivatives of 2-aminobenzoxazole **3**−**15** were prepared through a three-step synthetic route (Scheme **1**). The first step involved the cyclization of 2-aminobenzoxazole **1** by reacting 2-aminophenol with di(imidazol-1-yl)methanimine. In the second step, the resulting 2-aminobenzoxazole was propargylated using propargyl bromide without the addition of a base, as the presence of a base could lead to the formation of a dipropargylated derivative. This yielded the *N*-propargylated benzoxazole derivative **2**. In the final step, 1,2,3-triazole derivatives of 2-aminobenzoxazole **3**−**15** were synthesized *via* a Cu(I)-catalyzed click reaction between the *N*-propargylated benzoxazole derivative **2** and the corresponding azides. The Cu(I) catalyst was generated *in situ* from copper(II) sulfate pentahydrate and sodium ascorbate, which reduced Cu(II) to its catalytically active Cu(I) form. A slight excess of sodium ascorbate was used to prevent the reoxidation of Cu(I) to Cu(II), enabling the reaction to proceed efficiently without the need for an inert atmosphere.

**Scheme 2:**
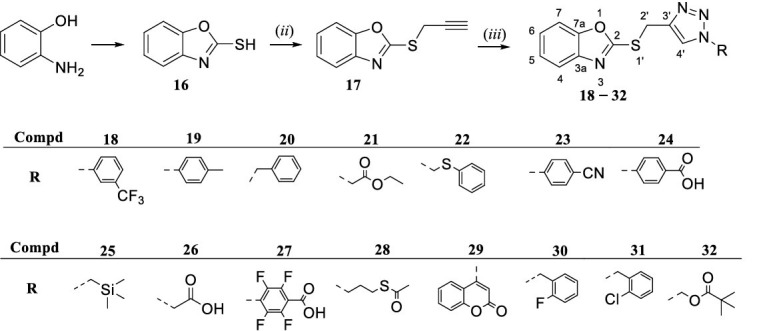
Reagents and conditions: (*i*) CS_2_, KOH, MeOH/H_2_O, reflux, (*ii*) prBr, K_2_CO_3_, acetone, RT, (*iii*) RN_3_, CuSO_4_ x 5 H_2_O, sodium ascorbate, *t*-BuOH/H_2_O.

Following a similar approach to the synthesis of 2-aminobenzoxazole derivatives, 1,2,3-triazole derivatives of 2-mercaptobenzoxazole **18**-**32** were prepared (Scheme **2**). The process began with a cyclization reaction of 2-aminophenol and carbon disulfide in the presence of KOH, yielding 2-mercaptobenzoxazole **16**. This intermediate was then converted into the S-propargylated derivative **17**, which subsequently underwent a Cu(I)-catalyzed click reaction with the corresponding azides to afford the desired 1,2,3-triazole derivatives of 2-mercaptobenzoxazole **18**-**32**.

The structures of the synthesized compounds were confirmed by one-dimensional 1H- and ^13^C-NMR spectroscopy, as well as mass spectrometry (Supplementary Fig. **S1**- Fig. **S93**). The successful completion of the click reaction and the formation of the 1,2,3-triazole ring in the benzoxazole derivatives were confirmed by the appearance of a proton signal 9 ppm in the ^1^H-NMR spectra, accompanied by the disappearance of the characteristic triplet associated with the terminal alkyne protons in the propargylated derivatives **2** and **17**. Additionally, in the ^13^C-NMR spectra, signals corresponding to the triazole ring carbons were observed at 130.50 ppm for C-3' and 122.48 ppm for C-4'.

### 4.2 *In Vitro* Antiproliferative Activity

All newly prepared compounds were tested for their *in vitro* antiproliferative activity on eight human cancer cells (LN-229 − glioblastoma, Capan-1 − pancreatic adenocarcinoma, HCT-116 − colorectal carcinoma, NCI-H460 − lung carcinoma, DND-41 − acute lymphoblastic leukemia, HL-60 − acute myeloid leukemia, K-562 − chronic myeloid leukemia, and Z-138 − non-Hodgkin lymphoma) using docetaxel and staurosporine as reference compounds. The results are expressed as IC_50_ values (50% inhibitory concentrations) and are shown in Table **1**. Benzoxazole derivatives substituted with a coumarin ring at the 4-position of the 1,2,3-triazole moiety, particularly compounds **3** and **29,** exhibited the most pronounced antiproliferative activity against all tested cell lines. The 2-mercaptobenzoxazole derivative **29** demonstrated significantly better activity compared to the 2-aminobenzoxazole derivative **3**. Compound **29** showed remarkable activity across all tested cell lines (IC_50_ = 1.0-2.1 µM), with its strongest effect against acute lymphoblastic leukemia (DND-41, IC_50_ = 1.0 µM). Compound **3** displayed the highest activity against glioblastoma (LN-229, IC_50_ = 6.7 µM) and pancreatic adenocarcinoma (Capan-1, IC_50_ = 6.5 µM), while also showing moderate efficacy against other tested cancer cell lines. Additionally, compound **13**, featuring a benzyl azide pharmacophore, exhibited moderate activity across all tested lines (IC_50_ = 36.2-57.4 µM). However, the majority of compounds showed little to no activity against the tested cell lines.

### 4.3 ADME Properties

The Absorption, Distribution, Metabolism, and Excretion (ADME) properties of the 1,2,3-triazole derivatives that exhibited antiproliferative activity are presented in Table **2**. According to Lipinski’s “Rule of Five,” which outlines physicochemical parameters indicative of high oral bioavailability, the following criteria should be met: a molecular weight between 150 and 500 g/mol, a fraction of sp^3^-hybridized carbons of at least 0.25, fewer than 9 rotatable bonds, a lipophilicity (XLOGP3) between -0.7 and +5.0; a Topological Polar Surface Area (TPSA) between 20 and 130 Å^2^; and a water solubility (log S) greater than 6 [69].

Among the compounds exhibiting antiproliferative activity, only derivatives **28** and **32** meet all drug-likeness criteria. The remaining ten compounds fail to satisfy one parameter, specifically the ratio of sp^3^-hybridized carbons to the total carbon count, which falls below 0.25, or they lack a sufficient number of saturated carbon-carbon bonds. In compound **28**, the ethanethioate group, and in compound **32**, the pivalate group, increase molecular saturation, which is positively correlated with solubility, a crucial factor influencing the bioavailability of potential drug candidates [70]. However, these two compounds were either completely inactive (compound **32**) or only moderately active (compound **28**) against the Capan-1, DND-41, HL-60, and Z-138 cell lines (Table **1**). An effective drug candidate should readily diffuse through several semipermeable cell membranes, enabling movement from gastrointestinal fluids into the bloodstream. The diffusion rate is influenced by the molecule's lipid solubility, size, degree of ionization, and the surface area available for absorption [71]. The Blood-Brain Barrier (BBB) serves as both a physical and biochemical shield, protecting the brain from peripheral substances while regulating the distribution of centrally acting compounds into the brain's bloodstream [72]. To predict drug absorption, a key pharmacokinetic property, we calculated the potential for passive Human Gastrointestinal Absorption (HGA) and BBB permeability using the Egan computational model [73]. The two most potent benzoxazole derivatives bearing a coumarin core, compounds **3** and **29**, demonstrated high potential for passive gastrointestinal absorption but a low likelihood of BBB penetration, thereby reducing the risk of unwanted central nervous system side effects.

**Fig. (3):**
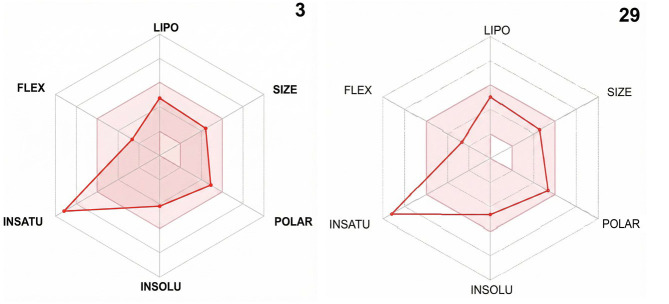
Bioavailability radars for 1,2,3-triazole derivatives **3** and **29**. The pink region denotes the optimal range for each property (lipophilicity (XLOGP3); size (MW); polarity (TPSA); water solubility (log S); saturation (Fraction Csp^3^); and flexibility (number of rotatable bonds).

The bioavailability radar schematically illustrates the drug-likeness properties of compounds **3** and **29**, which exhibited the most promising antiproliferative activity (Fig. **3**). These compounds deviate only from the saturation criterion due to a lack of sp^3^-hybridized carbon atoms in their structures. The ratio of sp^3^-hybridized carbons to the total number of carbon atoms is less than 0.25. While aromatic features can facilitate π-π interactions or π-cation interactions with proteins, saturated carbon-carbon bonds improve the compound’s ability to dock to the target protein.

### 4.4 Molecular Docking Studies

Mitogen-Activated Protein Kinases (MAPKs) transmit signals from the cell surface to the nucleus, regulating various cellular functions. TAO2, a MAP3K-level kinase, serves as an entry point into the MAPK signaling pathway. It is considered a potential drug target because it activates p38 MAPKs, which regulate inflammatory cytokines associated with tumor necrosis, inflammation, and immunoresponsive diseases. TAO kinases 1 and 2 are also potential targets for cancer therapy, as they regulate microtubule dynamics and organization [59, 74]. To explore the binding interactions of twelve benzoxazole derivatives, a molecular docking study was conducted using the ATP-binding cleft of TAO2. The TAO2 kinase domain binding site was defined based on the co-crystallized ligand staurosporine (PDB: 2GCD). Compounds were ranked according to the total energy of their predicted binding poses (Table **3**), and their docking scores were compared with those of staurosporine.

The most potent inhibitor of the TAO2 kinase domain, following the reference ligand staurosporine, is the 2-aminobenzoxazole derivative **3**. This compound exhibited excellent antiproliferative activity across nearly all tested cell lines, with the most pronounced effect observed against the pancreatic adenocarcinoma cell line (Capan-1). Recent studies have also indicated that staurosporine induces apoptosis in pancreatic carcinoma cells through the intrinsic signaling pathway [75]. The interaction energies between compound **3** and the residues in the TAO2 kinase binding site are presented in Table **4**, while visualizations of these interactions are shown in Fig. (**4**). The positioning of compound **3** within the binding site of the TAO2 kinase domain, depicted as a hydrophobic surface, is illustrated in Fig. (**5**).

Similar to staurosporine, compound **3** is positioned within the hydrophobic surface of the ATP-binding cleft of TAO2, engaging in multiple van der Waals interactions with surrounding residues. These include π-anion interactions with Asp114, π-donor hydrogen bonds with Asp169, π-σ interactions with Ile34, and π-alkyl interactions with Ala55, Lys57, Ile89, Met105, Lys314, and Leu138. The hydrogen bonding pattern of compound **3** is similar to that observed with staurosporine: one hydrogen bond is formed between a nitrogen atom of the 1,2,3-triazole moiety and Cys108, while a second hydrogen bond is established between the coumarinyl oxygen atom and Tyr107 [40].

Although the 2-mercaptobenzoxazole derivative **29** exhibited significantly better activity than the 2-aminobenzoxazole derivative **3** across all tested cell lines, particularly against acute lymphoblastic leukemia (DND-41, its interaction with TAO2 was found to be much weaker than that of compound **3**. Consequently, a molecular docking study was conducted using the tyrosine protein kinase c-Src (PDB ID: 3G6H) as an alternative target. c-Src is a proto-oncogene enzyme essential for regulating cell morphology, motility, proliferation, and survival, with its activity notably associated with the development of leukemia [76]. The binding site of c-Src was defined using the co-crystallized inhibitor DSA1 (PDB: 3G6H) [58]. The docking results for the twelve most active compounds, along with staurosporine and the bound ligand pyridinyl triazine (DSA1), are summarized in Table **5**.

The highest binding energy was observed for the complex with the inhibitor DSA1, which exhibited a binding energy of -133.74 kcal/mol. This was followed by the 2-mercaptobenzoxazole derivative **29**, with a binding energy of -131.44 kcal/mol, aligning with the experimental results presented in Table **1**. In contrast, staurosporine displayed a significantly lower binding energy of -110.18 kcal/mol, correlating with its moderate inhibitory effect against the acute lymphoblastic leukemia cell lines (DND-41). The energies of the main interactions between compound **29** and the residues in the c-Src binding site are detailed in Table **6** and visualized in Fig. (**6**).

Fig. (**7**) illustrates the optimal positioning of compound **29** within the hydrophobic binding pocket of c-Src. The ATP-binding site of the tyrosine kinase domain−crucial for the activity of small-molecule inhibitors such as imatinib, a well-established drug for chronic myeloid leukemia is located in a deep cleft between the two lobes of the tyrosine kinase domain. Compound **29** forms key hydrogen bonds with Tyr382 through two oxygen atoms from its coumarinyl group. The phenyl ring of the coumarinyl substituent also engages in π-σ interactions with Val402 and Leu322. Additionally, the 1,2,3-triazole ring participates in both π-σ and π-alkyl interactions with Met314 and Ala403. Further significant van der Waals interactions are established between the benzoxazole ring and residues Leu273, Val281, Ala293, Ile338, and Leu393.

Docking studies of azacridine derivatives revealed that the amido moiety forms two hydrogen bonds with Glu310 and Asp404, along with three π-π interactions and one cation-π interaction involving Leu273, Phe405, Tyr382, and Lys295, respectively [77]. Quinoline-arylamidine hybrids, which exhibit strong efficacy against human chronic myeloid leukemia and lymphoblastic cell lines, also dock within the c-Src binding site, forming hydrogen bonds between amino groups of the 2-aminoethanol linker and residues Asp404 and Tyr382. Additionally, the quinoline nitrogen atom interacts with Tyr382, and the nitrile group’s nitrogen forms a bond with Tyr340 [78].

## CONCLUSION

In this study, it is reported that the synthesis and the evaluation of antiproliferative activities of novel 2- substituted benzoxazole derivatives, featuring various substituents at position 4 of the 1,2,3-triazole ring. The novel 1,2,3-triazole derivatives of 2-aminobenzoxazole **3**−**15** and 2-mercaptobenzoxazole **18**−**32** were synthesized by applying a copper-catalyzed click reaction between propargylated benzoxazole derivatives and the corresponding azides. All newly synthesized compounds were tested on eight human cancer cell lines (LN-229, Capan-1, HCT-116, NCI-H460, DND-41, HL-60, K-562, and Z-138) using docetaxel and staurosporine as reference compounds. The most active and prominent derivatives were compounds **3** and **29**, both of which featured a coumarin ring at the 4-position of the 1,2,3-triazole moiety. These compounds exhibited inhibitory activity against all tested cancer cell lines. Notably, the 2-mercaptobenzoxazole derivative **29** demonstrated superior activity compared to the 2-aminobenzoxazole derivative **3** (IC_50_ = 6.5−76.2 µM). Compound **29** exhibited the most pronounced antiproliferative activity against all tested cell lines (IC_50_ = 1.0−2.1 µM), with particularly strong efficacy against acute lymphoblastic leukemia (DND-41, IC_50_ = 1.0 µM). The results suggest that both the nature of the substituents on the 1,2,3-triazole moiety and the type of linker between the benzoxazole and triazole rings significantly influence antiproliferative activity.

Both highly active compounds are promising candidates for drugs. Each violates only one drug-likeness criterion (the lack of sp^3^ hybridized carbon atoms), while meeting optimal criteria for lipophilicity, molecular weight, polarity, water solubility, and number of rotatable bonds. Furthermore, they show a high potential for passive gastrointestinal absorption and a low likelihood of crossing the blood-brain barrier. Molecular docking studies further support the antiproliferative activity results. The coumarinyl oxygen atom facilitates hydrogen bond formation, while the phenyl ring of the coumarin moiety engages in van der Waals interactions with amino acid residues in the target protein, similar to that observed with staurosporine. Molecular docking highlights the 2-aminobenzoxazole derivative **3** as a potential inhibitor of TAO2 kinase, which is associated with pancreatic adenocarcinoma, and the 2- substituted benzoxazole derivative **29** as a potential tyrosine-protein kinase inhibitor, exhibiting a binding mode similar to that of a highly effective drug used in the treatment of chronic myeloid leukemia. The presented results indicate that this class of 2-amino and 2-mercaptobenzoxazoles bearing a 1,2,3-triazole moiety can be effectively synthesized using click chemistry and represent promising candidates for further design and optimization toward the development of potent antiproliferative agents.

## STUDY LIMITATIONS

This research was limited to a relatively small set of 2-arylbenzoxazole derivatives evaluated mainly through *in vitro* assays and docking models, without *in vivo* validation or detailed pharmacokinetic assessment. Therefore, broader screening and further ADME studies are needed to confirm their therapeutic potential.

## Figures and Tables

**Fig. (4):**
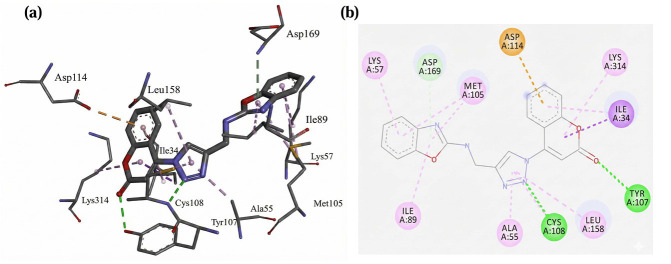
The main interactions between compound **3** and the residues of the TAO2 kinase. (green = conventional hydrogen bond; light green = π-donor hydrogen bond; brown = π-anion; purple = π-σ interactions; pink = π-alkyl interactions; brown = π-kation): **a**) 3D, and **b**) 2D representation.

**Fig. (5):**
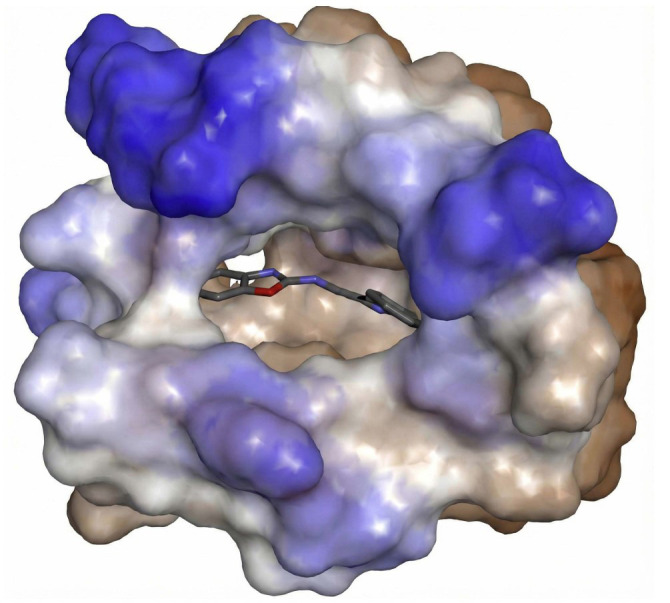
Hydrophobic surface representation of the ATP-binding cleft of TAO2 with docked compound **3**. (Hydrophobicity range: brown = 3; white = 0; blue = -3).

**Fig. (6):**
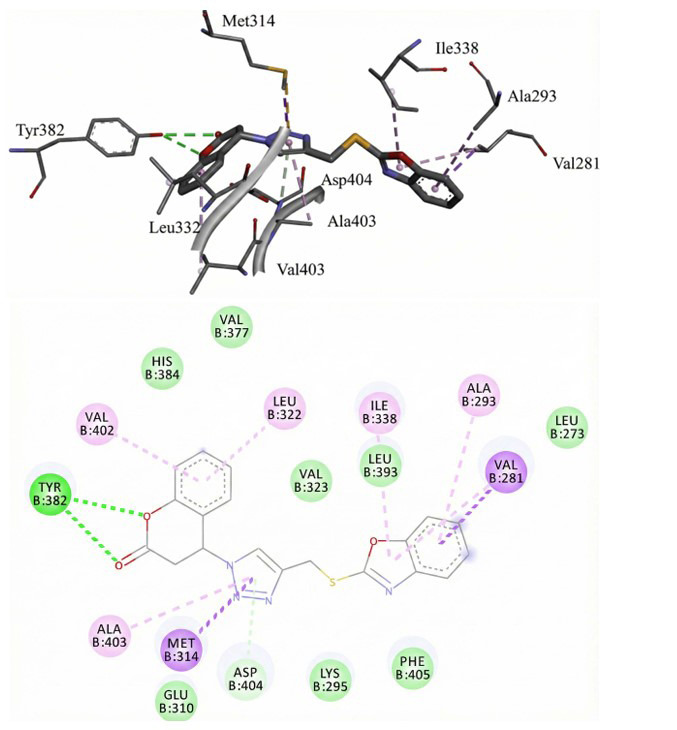
The main interactions between compound **29** and residues of the protein-tyrosine kinase (c-Src). (green = conventional hydrogen bond; light green = π-donor hydrogen bond; brown = π-anion; purple = π-σ interactions; pink = π-alkyl interactions; brown = π-kation): a) 3D, and b) 2D representation.

**Fig. (7):**
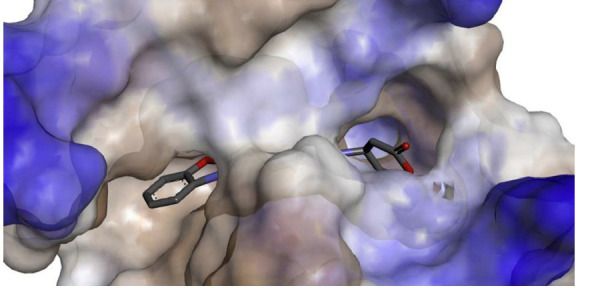
Hydrophobic surface representation of the ATP-binding cleft of c-Src with docked compound **29**. (Hydrophobicity range: brown = 3; white = 0; blue = -3).

**Table 1:** Antiproliferative activity of the 1,2,3-triazole derivatives of 2-aminobenzoxzazole **3**−**15** and 2-mercaptobenzoxazole **18**−**32.**

**Compd**	**Concentration Unit**	**IC_50_**							
		**LN-229 Glioblastoma **	**Capan-1 Pancreatic Adenocarcinoma **	**HCT-116 Colorectal Carcinoma **	**NCI-H460 Lung Carcinoma **	**DND-41 Acute Lymphoblastic Leukemia **	**HL-60 Acute Myeloid Leukemia **	**K-562 Chronic Myeloid Leukemia **	**Z-138 non-Hodgkin lymphoma **
**3**	µM	6.7±3.1	6.5±3.8	24.2±1.6	30.5±3.3	13.0±0.8	48.7±0.4	76.2±11.7	12.6±0.4
**4**	µM	>100	>100	>100	>100	>100	>100	>100	>100
**5**	µM	>100	>100	>100	64.8	>100	>100	>100	>100
**6**	µM	>100	≥32.9	≥52.2	49.8±2.6	>100	≥97.4	>100	≥90.5
**7**	µM	≥45.9	≥42.8	>100	>100	>100	>100	>100	≥85.5
**8**	µM	>100	≥81.8	>100	>100	>100	>100	>100	>100
**9**	µM	>100	>100	>100	>100	>100	>100	>100	>100
**10**	µM	>100	>100	>100	>100	>100	>100	>100	>100
**11**	µM	>100	>100	>100	>100	>100	>100	>100	>100
**12**	µM	>100	>100	>100	>100	>100	>100	>100	>100
**13**	µM	47.7±6.1	49.0±2.3	50.8±8.1	36.2±0.6	53.8±2.4	40.6±0.6	≥53.3	57.4±4.0
**14**	µM	>100	>100	>100	>100	>100	>100	>100	>100
**15**	µM	59.45±7.8	54.2±1.7	60.3±1.5	18.5±8.1	71.2±1.3	52.4±5.2	>100	58.3±2.1
**18**	µM	>100	>100	>100	>100	>100	>100	>100	>100
**19**	µM	>100	>100	>100	>100	>100	>100	>100	>100
**20**	µM	>100	>100	>100	>100	>100	>100	>100	>100
**21**	µM	>100	>100	>100	>100	>100	>100	>100	>100
**22**	µM	>100	>100	>100	>100	>100	>100	>100	>100
**23**	µM	>100	>100	>100	≥57.9	>100	>100	>100	>100
**24**	µM	>100	≥42.9	>100	≥60.9	>100	67.0±9.5	>100	≥58.3
**25**	µM	>100	>100	>100	>100	>100	>100	>100	>100
**26**	µM	>100	>100	>100	>100	>100	>100	>100	>100
**27**	µM	>100	≥48.8	>100	>100	>100	≥95.9	>100	>100
**28**	µM	>100	52.6±0.8	>100	>100	54.5±9.0	47.0±1.9	>100	54.5±2.1
**29**	µM	1.8±0.5	1.3±0.1	1.4±0.4	2.1±0.2	1.0±0.9	1.4±0.1	1.6±1.1	1.8±0.4
**30**	µM	>100	>100	>100	>100	>100	>100	>100	>100
**31**	µM	>100	>100	>100	>100	>100	>100	>100	>100
**32**	µM	>100	>100	>100	>100	>100	>100	>100	>100
Docetaxel	nM	4.1±2.7	3.8±2.6	2.5±0.4	3.4±0.2	2.5±0.1	2.2±0.4	8.5±0.9	2.3±0.1
Staurosporine	nM	66.8±9.0	51.9±7.4	70.1±5.9	44.8±4.0	54.8±1.1	58.6±3.5	37.4±5.9	48.4±7.2

**Table 2:** ADME properties of the most active 1,2,3-triazole derivatives.

**Compd**	**MW**	**Fract. Csp^3^**	**RB**	**HBA**	**HBD**	**TPSA**	**XLOGP3**	**Silicos-IT LogSw**	**Silicos-IT class**	**HGI absorption.**	**BBB perm.**
**3**	359.34	0.05	4	6	1	98.98	2.74	-7.21	Poorly soluble	High	No
**6**	325.75	0.06	4	4	1	68.77	3.66	-6.64	Poorly soluble	High	Yes
**7**	335.32	0.06	5	6	2	106.07	2.56	-5.39	Moderately soluble	High	No
**8**	370.2	0.06	4	4	1	68.77	3.73	-6.84	Poorly soluble	High	Yes
**13**	305.33	0.12	5	4	1	68.77	2.97	-6.44	Poorly soluble	High	Yes
**15**	359.31	0.12	5	7	1	68.77	3.92	-6.89	Poorly soluble	High	Yes
**23**	347.39	0.11	5	5	0	105.83	3.22	-6.56	Poorly soluble	High	No
**24**	352.37	0.06	5	6	1	119.34	3.09	-5.44	Moderately soluble	High	No
**27**	424.33	0.06	5	10	1	119.34	3.49	-6.5	Poorly soluble	Low	No
**28**	348.44	0.33	8	5	0	124.41	2.63	-5.21	Moderately soluble	High	No
**29**	376.39	0.05	4	6	0	112.25	3.27	-7.26	Poorly soluble	High	No
**32**	346.4	0.38	7	6	0	108.34	3.23	-4.85	Moderately soluble	High	No

**Note: **MW (molecular weight); Fract. Csp^3^ (Fraction Csp^3^); RB (number of rotatable bonds); HBA (number of hydrogen-bond acceptors; HBD (number of hydrogen-bond donors); TPSA (topological polar surface area); XLOGP3 (lipophilicity descriptor); Silicos-IT LogSw (logarithm of the molar solubility in water); HGI absorp. (human passive gastrointestinal absorption; BBB perm (blood-brain barrier permeation).

**Table 3:** Binding energies (kcal/mol) of 1,2,3-triazole derivatives and standard ligand staurosporine (STU) on TAO2 kinase domain (PDB ID: 2GCD).

**Compound (pose)**	**Total energy**	vdW*	**HBond**	**Elec**
**STU** (2)	-137.90	-123.90	-14.00	0.00
**3** (2)	-113.74	-108.96	-4.78	0.00
**27** (2)	-110.42	-83.54	-25.01	-1.86
**7** (1)	-109.04	-83.78	-25.26	0.00
**24** (0)	-108.43	-91.91	-20.89	4.37
**29** (2)	-107.74	-82.68	-25.06	0.00
**15** (2)	-105.03	-84.20	-20.83	0.00
**32** (2)	-103.44	-74.54	-28.89	0.00
**9** (2)	-100.76	-83.21	-17.56	0.00
**6** (2)	-99.16	-72.05	-27.11	0.00
**12** (2)	-97.70	-69.38	-28.33	0.00
**8** (1)	-95.92	-71.95	-23.97	0.00
**7** (1)	-92.58	-54.20	-38.38	0.00

**Note: ** *Interactions: vdW (van der Waals); Hbond (hydrogen-bonding); Elec (electrostatic).

**Table 4:** The energies (kcal/mol) of the main interactions between the TAO2 kinase domain and compound **3.**

**H Bond**	**Energy**	**Van der Waals Interaction**	**Energy**
M-Glu106	-2.28	M-Ile34	-4.34
S-Cys108	-2.50	S-Ile34	-11.23
-	-	M-Gly35	-0.75
-	-	S-Phe39	-0.83
-	-	S-Val42	-2.34
-	-	S-Lys57	-5.10
-	-	S-Ile89	-6.38
-	-	S-Met105	-4.94
-	-	M-Tyr107	-7.93
-	-	M-Cys108	-2.96
-	-	M-Gly110	-5.53
-	-	M-Ser111	-1.85
-	-	S-Asp114	-3.36
-	-	M-Gly155	-0.34
-	-	S-Leu158	-8.05
-	-	M-Gly168	-4.22
-	-	M-Asp169	-6.82
-	-	S-Asp169	-6.06
-	-	S-Lys314	-5.07

**Note: **(M = main chain; S = side chain).

**Table 5:** Binding energies (kcal/mol) of 1,2,3-triazole derivatives and standard ligand staurosporine (STU) on tyrosine-protein kinase c-Src (PDB ID: 3G6H).

**Compound (pose)**	**Total energy**	vdW*	**HBond**	**Elec**
**DSA1**(2)	-133.74	-120.60	-13.14	0.00
**29** (2)	-131.44	-109.10	-22.34	0.00
**7** (2)	-125.50	-105.93	-18.66	-0.91
**15** (1)	-122.49	-110.34	-12.15	0.00
**27** (2)	-120.61	-98.33	-21.99	-0.29
**32** (0)	-117.84	-82.69	-35.15	0.00
**28** (2)	-117.67	-85.36	-32.31	0.00
**13** (0)	-116.52	-96.16	-20.36	0.00
**24** (0)	-114.49	-97.14	-16.31	-1.03
**23** (0)	-110.33	-92.47	-17.86	0.00
**STU** (1)	-110.18	-103.65	-6.53	0.00
**3** (2)	-106.65	-92.29	-14.36	0.00
**8** (0)	-106.64	-94.69	-11.94	0.00
**6** (0)	-105.13	-94.31	-10.82	0.00

Note: *Interactions: vdW (van der Waals); (Hbond) hydrogen-bonding; (Elec) electrostatic.

**Table 6:** The energies (kcal/mol) of the main interactions between the residues of the binding site of the c-Src and compound **29**.

**H Bond**	**Energy**	**Van der Waals Interaction**	**Energy**
S-Glu310	-7.00	S-Leu273	-1.40
S-Tyr382	-4.98	S-Val281	-4.98
M-Asp404	-7.95	S-Lys295	-3.02
S-Asp404	-2.40	M-Glu310	-0.84
-	-	S-Glu310	-5.96
-	-	S-Met314	-8.63
-	-	S-Leu322	-3.40
-	-	S-Val323	-3.50
-	-	S-Ile338	-3.54
-	-	S-Tyr382	-6.50
-	-	S-His384	-5.26
-	-	S-Leu393	-3.11
-	-	M-Val402	-3.34
-	-	M-Ala403	-10.05
-	-	M-Asp-404	-11.84
-	-	S-Asp404	-7.71
-	-	M-Phe405	-2.70
-	-	S-Phe405	-8.52
-	-	M-Gly406	-0.12

**Note: **(M = main chain; S = side chain).

## Data Availability

The data and supportive information are available within the article.

## LIST OF ABBREVIATIONS

HGA: Human Gastrointestinal Absorption
BBB: Blood-Brain Barrier
NSAID: Nonsteroidal Anti-Inflammatory Drug
CuAAC: Copper-catalyzed Azide-alkyne Cycloaddition
ADME: Absorption, Distribution, Metabolism, and Excretion
